# A new chitinozoan assemblage from the Middle Devonian Los Monos Formation (sub-Andean basin, southern Bolivia) and its biozonal implications for Western Gondwana

**DOI:** 10.1371/journal.pone.0297233

**Published:** 2024-04-09

**Authors:** Sonia C. Camina, Claudia V. Rubinstein, Anthony Butcher, Victoria J. García Muro, Gustavo Vergani, Martín Pereira

**Affiliations:** 1 CCT CONICET Mendoza, IANIGLA, Grupo de Paleopalinología y Paleoecología Vegetal, Mendoza, Argentina; 2 University of Portsmouth, School of the Environment, Geography and Geosciences, Portsmouth, United Kingdom; 3 Pluspetrol S.A., CABA, Buenos Aires, Argentina; CNRS-University of Lille, FRANCE

## Abstract

Chitinozoans recovered from one section of the Middle Devonian Los Monos Formation in the TCB X-1001-Tacobo borehole, sub-Andean basin of Bolivia, have been analysed. Eleven from the eighteen processed cutting samples yielded specimens that allowed taxonomic study. Eleven genera and thirty-five chitinozoan species were identified from the Los Monos Formation with four of them recorded for the first time in Western Gondwana. *Ancyrochitina biconstricta*, *Ancyrochitina parisi*, *Angochitina galarzae* and *Ramochitina boliviensis* are among the most relevant taxa restricted to Western Gondwana that support the affinity with this paleocontinent. One new species, *Lagenochitina tacobensis* sp. nov. is described, and *Ramochitina candelariaensis* sp. nov. (n. n.) is formally erected. The chitinozoan assemblage reinforces the late Eifelian–middle Givetian age previously proposed by organic-walled phytoplankton and miospores for this section of the TCB X-1001-Tacobo borehole. A new local chitinozoan biozonation based on the chitinozoan assemblages is proposed and a revision of the current chitinozoan biozonation for Western Gondwana and Bolivia is recommended.

## 1. Introduction

The sub-Andean basin of southern Bolivia constituted part of Western Gondwana throughout the Devonian. The geological evolution of this Gondwana margin during the Phanerozoic is defined by a tectonically active and structurally complex framework that allowed the sedimentary infill to be divided into several overlapping sedimentary basins [1]. One of these basins developed during the Silurian through the Devonian with a large volume of siliciclastic sediments deposited by relative sea-level changes. The vastness and complex tectonic evolution of these Phanerozoic basins have made biostratigraphical and palynological studies extremely important to understand the stratigraphical configuration of Devonian sediments.

Most of the previous biostratigraphical studies in these basins are based on palynological data due to the scarcity of other useful fossil groups [2]. The complete list of previous palynological studies in the area can be found in García Muro et al. [3].

The TCB X-1001-Tacobo borehole was drilled in the eastern sector of the sub-Andean ranges known as ‘the Foothills’. The samples studied come from the Icla, Huamampampa and the Los Monos formations. The phytoplankton and miospores were described by García Muro et al. [3], thus the present study focuses on the chitinozoans from the Los Monos Formation, which is the other important palynological marine group.

Chitinozoan studies are very important in Palaeozoic marine strata, as they provide excellent data for detailed biostratigraphical studies, together with phytoplankton and miospores. The main advantages of this fossil group are their wide palaeogeographical distribution, rapid morphological evolution and the variety of marine sedimentary facies from which they can be recovered (e.g. [4–6]).

Since previous records of detailed chitinozoan taxonomy from the Los Monos Formation in Bolivia are extremely scarce [7–9], this work aims to provide a new fully-detailed record of their taxonomy in order to contribute to a better knowledge of the Devonian chitinozoan assemblages of Western Gondwana.

Another important goal of this research is to accurately constrain the age of the Los Monos Formation in the Tacobo borehole, previously based on miospore and organic-walled phytoplankton assemblages. A chitinozoan Local Biozonation is proposed for the Los Monos chitinozoan assemblage to be compared with other coeval assemblages of South America and correlated to both the regional chitinozoan biozonations for Bolivia [9] and Western Gondwana [10].

## 2. Geological setting and stratigraphy

**Fig 1:**
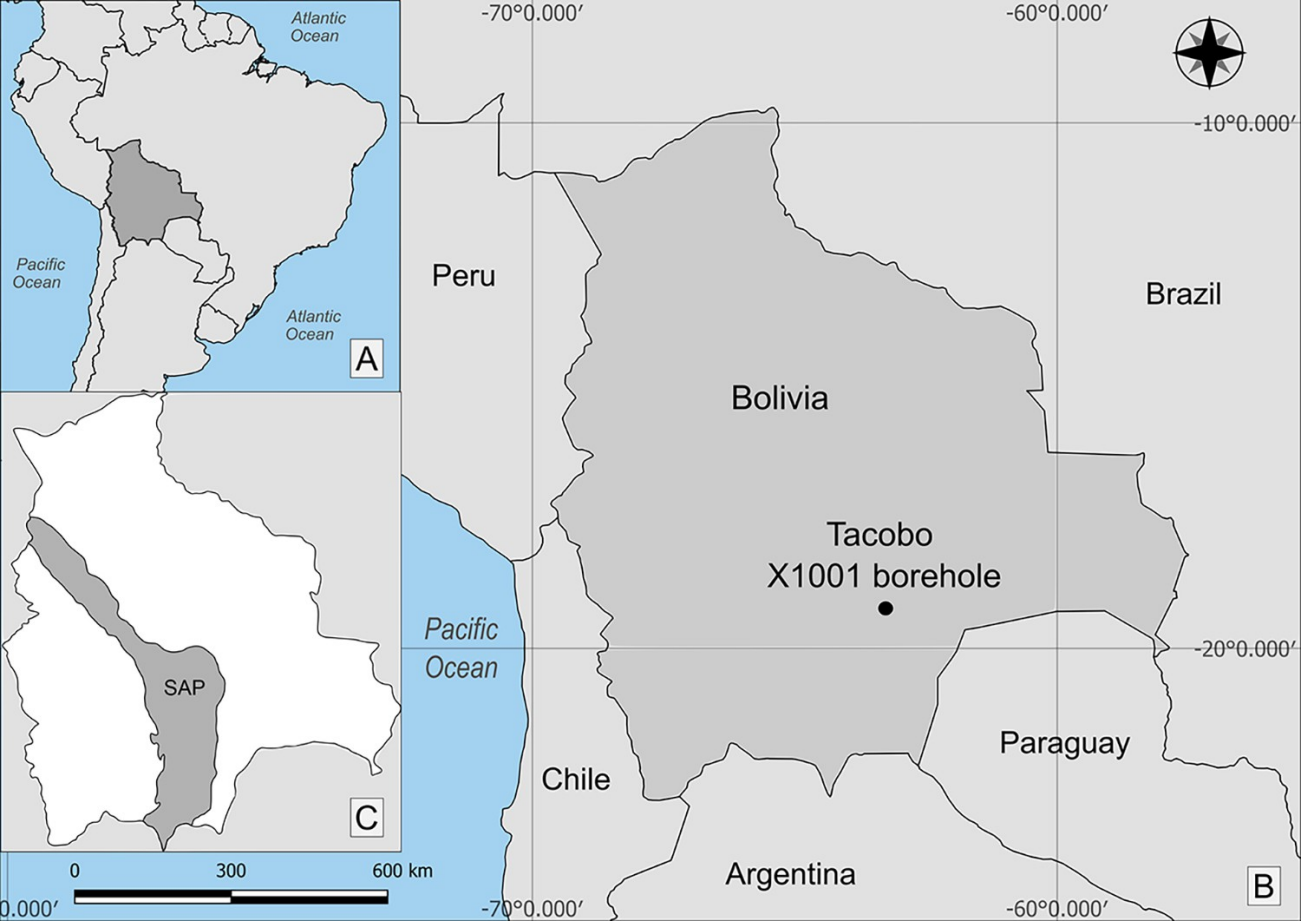
Location map of the Tacobo X1001 borehole. A: Location map of Bolivia in South America. B: Location map of the Tacobo borehole in Bolivia. C: Geological Province of the Subandino (SAP) of Bolivia.

The TCB X-1001-Tacobo borehole was drilled in the eastern sector of the sub-Andean ranges (the ‘Foothills’), in southern Bolivia (Fig 1). Previous palynological work on this well was published in detail by García Muro et al. [3] in which the marine deposits of the Icla, Huamampampa and Los Monos formations were analysed and the corresponding geology and stratigraphy were extensively described.

During the Devonian, the sub-Andean basin of Bolivia formed part of Western Gondwana and was situated about 60° S [2]. This high palaeolatitude and shallow marine deposits suggested cold sea conditions and a palaeobathymetry of no more than 200 m [11,12]. Although the middle and upper Palaeozoic tectonic setting along the Bolivian basins is still under debate (see [3]), the sedimentary infill is considered to be made up of several overlapping sedimentary basins.

**Fig 2:**
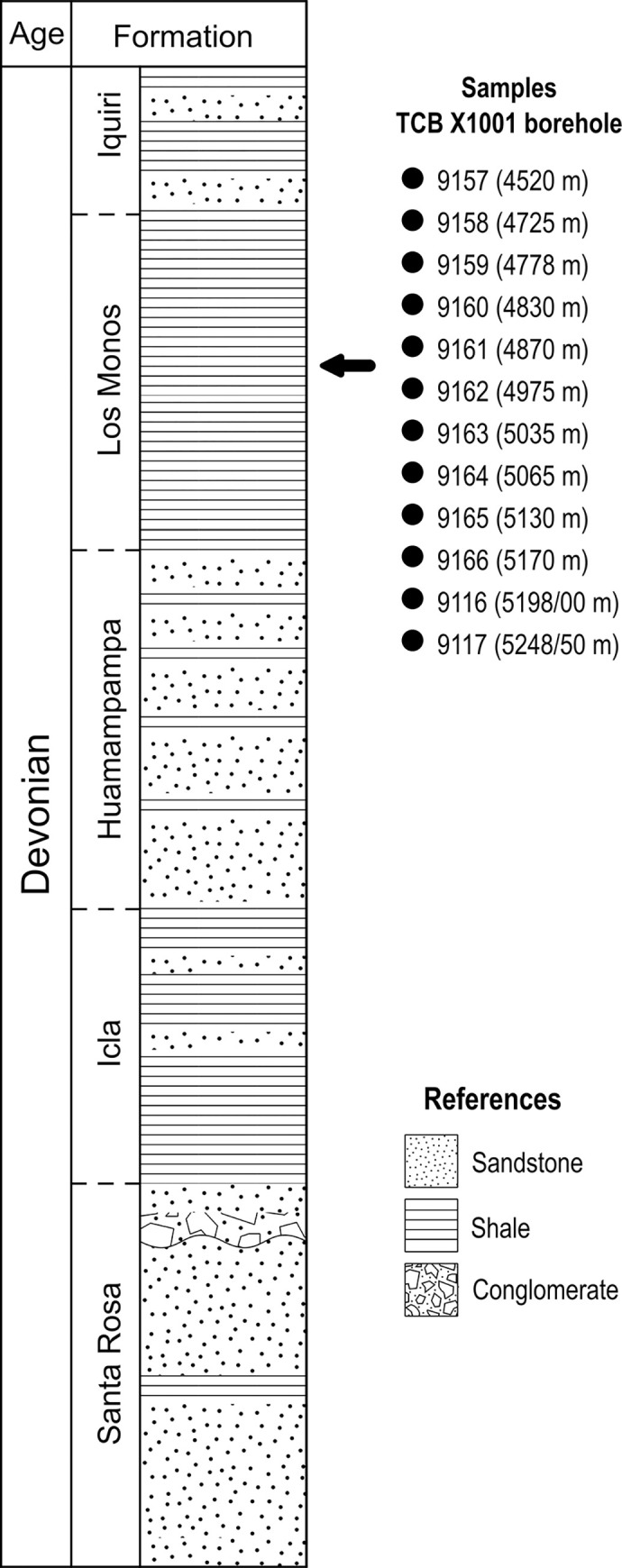
Schematic stratigraphic column of the TCB X-1001-Tacobo borehole. Depths of the Los Monos Formation palynological samples are indicated by meters below ground surface (mbgs) and the number of the sample.

The depositional system during the Silurian-Devonian was influenced by relative sea-level changes resulting in the deposition of a large volume of clastic sediments separated by flooding surfaces. These surfaces allowed Starck et al. [13,14] to propose three supersequences: Cinco Picachos, Las Pavas, and Aguaragüe. The Los Monos Formation (Fig 2) is the basal unit of the Aguaragüe supersequence [1,13–16] and is considered as the main source of oil and gas in the area.

The Los Monos Formation comprise dark grey to black laminated shales, with fine-grained and muddy sandstones [17]. These rocks were deposited mostly in a shallow to outer shelf marine palaeoenvironment and some local intervals seems to be nearshore or transitional with the overlying unit [18,19].

Previous studies dated the Los Monos Formation as late Eifelian [17,20], late Eifelian-late early Givetian to late Givetian [21] and early-middle Givetian to Frasnian [7]. García Muro et al. [3] have constrained the age of the Los Monos Formation, in the Tacobo borehole, to the late Eifelian?–middle Givetian, based on miospore and organic-walled phytoplankton assemblages.

## 3. Material and methods

Eighteen of the twenty-three cutting samples from the TCB X-1001-Tacobo borehole were processed for chitinozoan analysis. The samples of the Icla and Huamampampa formations, ranging from depths of 5710 to 5418 m, yielded scarce and poorly-preserved specimens which cannot be identified. Therefore, the chitinozoans from eleven samples of the Los Monos Formation were studied in detail (5248–4520 m), as these were generally well-preserved and more abundant.

The samples were processed at the Paleopalynology Laboratory of IANIGLA, CCT CONICET Mendoza, Argentina. No permits were required for the described study, which complied with all relevant regulations. 10 to 30 g of each sample were cleaned and processed using an HCl–HF–HCl acid maceration technique described in detail by Paris [22]. The organic residue was passed through 10 μm and 40 μm sieve meshes. The organic fraction retained in the 40 μm sieve was picked using a binocular microscope, and all chitinozoans specimens were extracted and mounted on 1 cm coverslips to be analysed using a scanning electron microscope (SEM). The cover slip was secured with an aluminium foil which was attached to the stubs using double-sided tape, and afterwards coated using gold metallization at 90% for 60 seconds. This thin coating allows samples to be mounted for subsequent observation in transmitted light if desired. Analysis was conducted using a JEOL JSM-6490LV SEM, at 5 kV.

**Fig 3:**
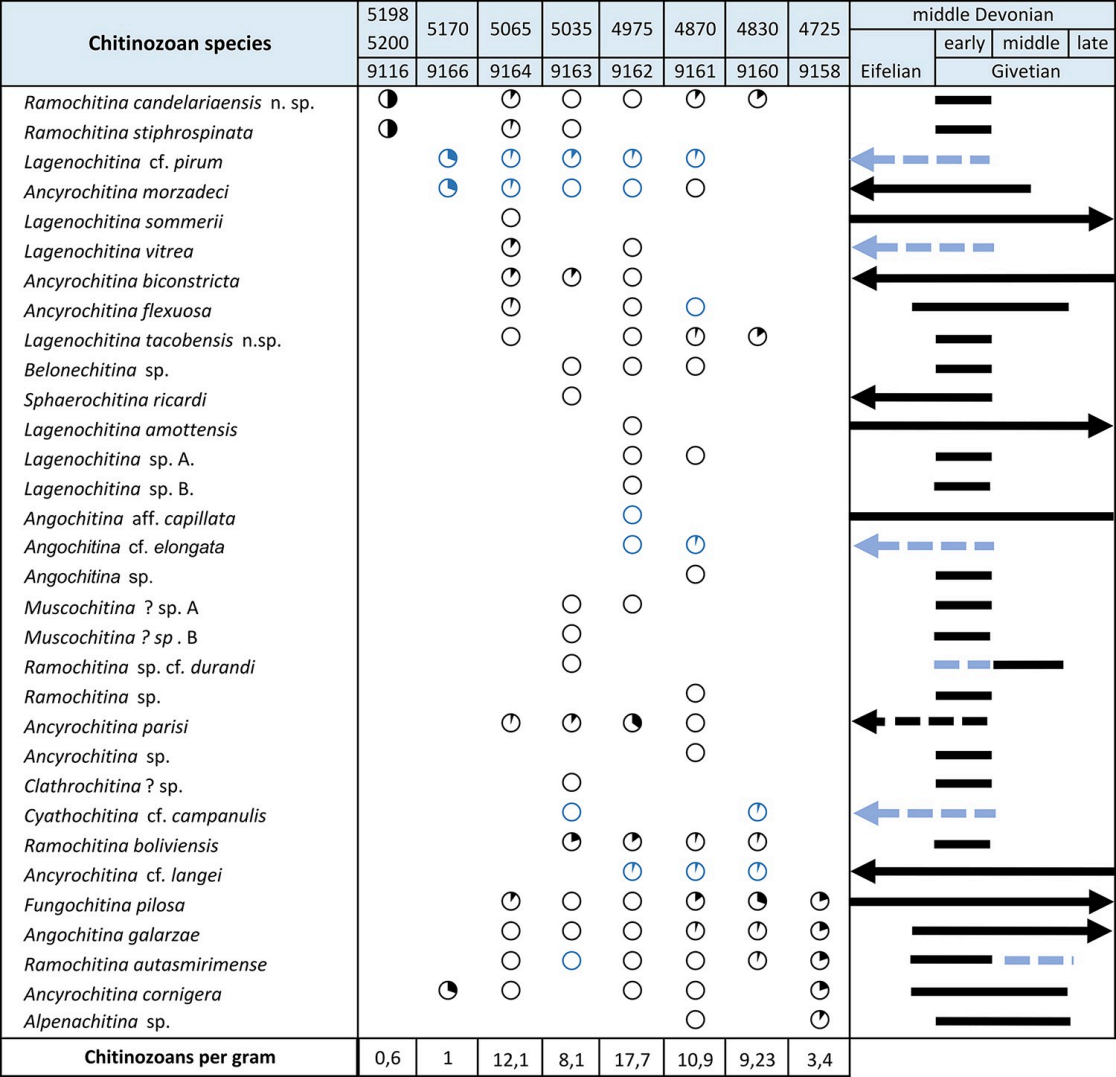
Stratigraphic distribution and abundances of chitinozoans. Black graphics indicate positively identified species and their relative abundance; blue graphic indicate open nomenclature species and their relative abundance. The stratigraphic range of each species is indicated with a straight line, and in dash line the possible extension of the stratigraphical range of the species.

Regarding the eleven studied samples, only eight yielded identifiable specimens allowing for taxonomic classification. All chitinozoans were counted to obtain absolute abundances ranging from 0.6 to 17.7 specimens per gram of rock (Fig 3). Pyrite was observed attached to some chitinozoan specimens distorting or breaking the walls of the chamber and/or the neck. The preservation was predominantly good, although all specimens were flattened and some of them were broken or lost processes and ornamentation (e.g. removal of the basal processes and/or long spines), hindering their identification.

The slides are housed in the palaeopalynological collection of IANIGLA, CCT CONICET Mendoza, Argentina.

## 4. Results

### 4.1. Chitinozoan assemblage

A total of eleven genera and thirty-five chitinozoan species were identified from the Tacobo borehole, with nineteen being retained in open nomenclature. Five of the taxa are recorded here for the first time in Western Gondwana, and nine are restricted to Western Gondwana—two new species are formally described herein.

The highest abundances (9.2 to 17.7 specimens per gram of rock) were recorded in the middle part of the studied interval of the Los Monos Formation, between 4830 to 5170 m depth (samples 9166 to 9160), particularly from depths 4870 and 4975 m (samples 9161 and 9162) with 19 and 21 species respectively. Meanwhile, in the upper and lower part, the diversity decreases to 5 and 2 chitinozoan species respectively. This relatively high chitinozoan abundance drops significantly in the uppermost and lowermost part of the studied section in samples 9116 and 9158 (0.6 to 3.4 specimens per gram of rock) (Fig 3).

### 4.2. Systematic palaeontology

The classification used herein follows the scheme proposed by Paris et al. [23], and the system of open nomenclature follows the recommendations of Bengtson [24].

The biometric measurements were taken in their raw form with no correction factor applied for flattening. Although our specimens are entirely compressed, raw data is adopted herein as correction factors are subjective and can be applied to the measurements herein by subsequent workers if desired.

The principal measurements, recorded in micrometres (μm), are: L = total length; ln = length of the neck; lc = length of the chamber; D = maximum diameter of the chamber; dn = diameter of the neck, and da = diameter of the aperture.

Group **Chitinozoa** Eisenack, 1931 [25]

Order **Prosomatifera** Eisenack, 1972 [26]

Family **Conochitinidae** Eisenack, 1931 [25] emend. Paris, 1981 [27]

Subfamily **Belonechitininae** Paris, 1981 [27]

Genus ***Belonechitina*** Jansonius, 1964 [28]

Type species: *Conochitina micracantha robusta* Eisenack, 1959 [29]

*Belonechitina* sp.

Fig 4A–4C.

**Fig 4:**
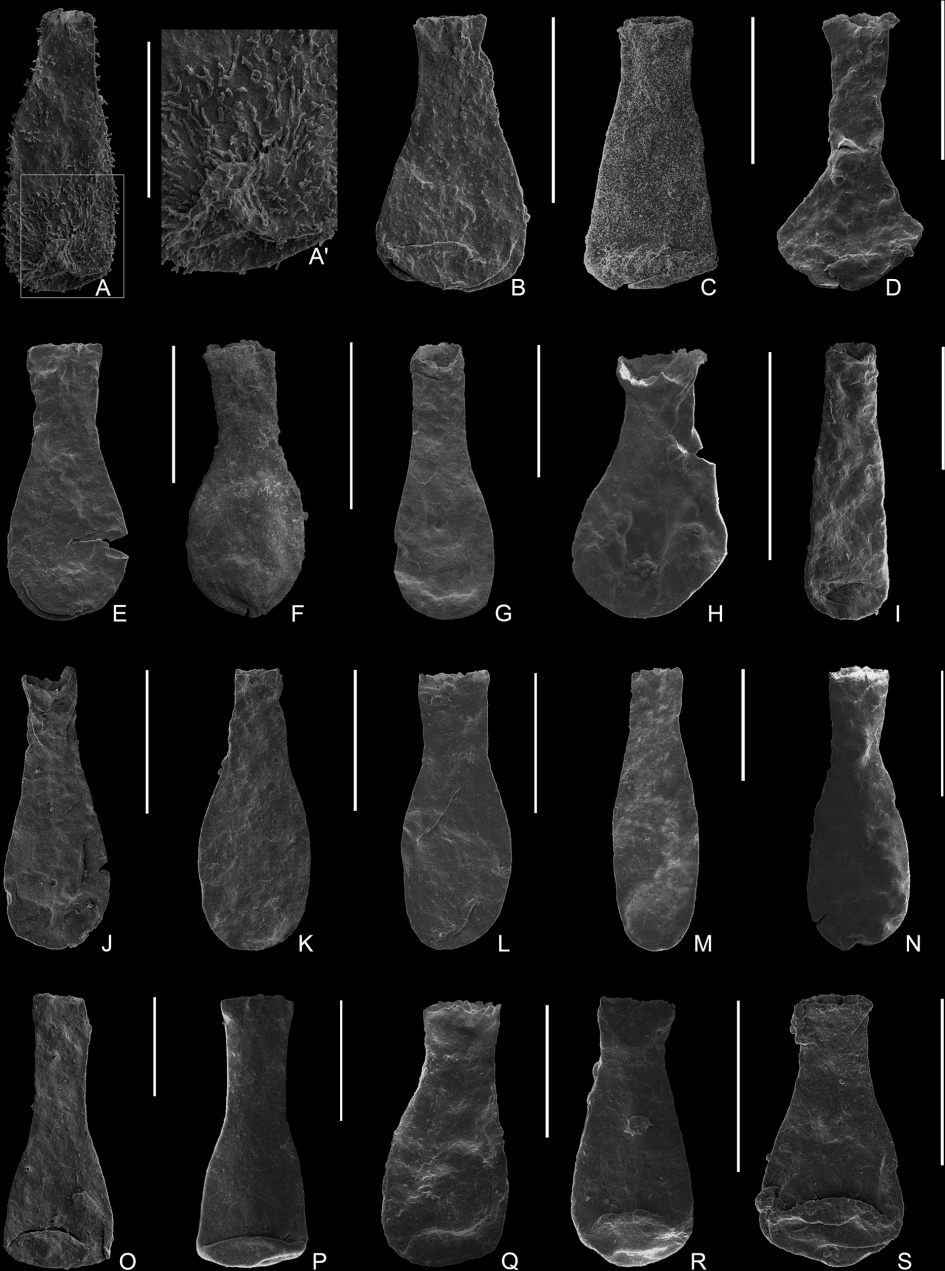
**Plate 1.** (A–C) *Belonechitina* sp., (A) 9163-S22-84, from Sample 9163; (A’) detail of the ornamentation showing randomly distributed granules and simple spines; (B–C) 9162-S13-57, 9162 -S16-78, from Sample 9162. (D) *Sphaerochitina ricardi* Díez & Cramer, 1978, 9163-S22-05, from Sample 9163. (E–G) *Lagenochitina amottensis* Grignani & Mantovani, 1964, 9162-S13-35, 9162-S7-34, 9162-S13-84, from Sample 9162. (H) *Lagenochitina sommerii* (Lange, 1952), 9164-S18-22, from Sample 9164. (I–M) *Lagenochitina tacobensis* sp. nov., (I, N) 9161-S19-30, from Sample 9161, (N) holotype 9161-S20-51, displaying an ovoid elongated vesicle chamber with short neck and smooth surface (J–L) 9162-S11-11, 9162-S13-04, 9162-S16-75, from Sample 9162; (M) 9164-S2-08, from Sample 9164; (O–P) *Sphaerochitina vitrea* (Taugourdeau, 1962), (O) 1962-S10-31, from Sample 1962; (P) 9164-S17-48, from Sample 9164. (Q–S) *Lagenochitina* cf. *pirum* (Achab, 1982), (Q) 9161-S20-02, from Sample 9161; (R–S) 9163-S22-25, 9163-S22-96, from Sample 9163. All scale bars represent 100 μm.

**Material**: Eleven specimens, four well-preserved and seven moderately well-preserved were observed and measured from samples 9161 (4870 m), 9162 (4975 m) and 9163 (5035 m) (Table 1).

**Table 1:** *Belonechitina* sp. measurements.

Dimensions (μm)	L	ln	lc	D	dn	da	%neck	fracc	L:D	lc:ln	D:dn	L:lc
Mean	183	53	130	79	41	42	30%	1/3	2:1	2:1	2:1	1:1
Min	141	35	91	68	34	34						
Max	250	63	189	90	49	50						

**Diagnosis**: Belonechitininae with cylindro-conical vesicle chamber and randomly distributed simple and very short spines and tubercles.

**Description**: The vesicle chamber is cylindro-conical, with a flat to slightly convex base and blunt basal margin. The flanks are straight with an inconspicuous to slight flexure and a very weak to absent shoulder. The neck is cylindrical and occasionally flares slightly towards the aperture, which occupies approximately one-third (30%) of the total length of the vesicle. Randomly distributed granules and simple spines (Fig 4A’) cover the whole vesicle with even density. The length of the ornamentation varies between 0.7 and 10 μm, and they are spaced between 0.3 and 1.7 μm apart on the vesicle surface. The basal features were only observed in one specimen, as the bases are flattened in all others, and no mucron or other distinguishing characteristics were observed.

**Remarks**: The specimens assigned to this taxon herein are retained in open nomenclature, but display the main characteristics of the genus *Belonechitina*; Conochitinidae with randomly distributed simple spines. Ottone [30] described *Belonechitina holfeltzii*, an Argentinean species, as a possible transition from the genus *Conochitina* to *Angochitina* which is similar in shape to our taxon. However, the ornamentation of *B*. *holfetzii* is comprised of spines branching distally, whereas our specimens have simple spines and tubercles.

**Occurrence**: *Belonechitina* sp. is recorded herein from the early Givetian in the TCB X-1001-Tacobo borehole.

Family **Lagenochitinidae** Eisenack, 1931 [25] emend. Paris, 1981 [27]

Subfamily **Lagenochitininae** Paris, 1981 [27]

Genus ***Sphaerochitina*** Eisenack, 1955 [31]

Type species: *Sphaerochitina sphaerocephala* Eisenack, 1955 [31]

*Sphaerochitina ricardi* Díez & Cramer, 1978 [32]

.1978 *Sphaerochitina ricardi*; Díez & Cramer, pp. 212–213, pl. 2, figs 75–79. [32]

.2019 *Sphaerochitina ricardi*; Askew & Russell, p. 80, pl. II, fig. 16; pl. III, fig. 7. [33]

Fig 4D.

**Type specimen:** Holotype: Díez & Cramer [32 pl. 2, fig. 79].

**Material**: One flattened and moderately well-preserved specimen was observed and measured from sample 9163 (5035 m) (Table 2).

**Table 2:** *Sphaerochitina ricardi* measurements.

Dimensions (μm)	L	ln	lc	D	dn	da	%neck	fracc	L:D	lc:ln	D:dn	L:lc
Mean	174	86	88	92	34	48	50%	1/2	2:1	1:1	3:1	2:1

**Description**: The vesicle chamber is sphero-conical, with a convex base and broadly rounded basal margin. The flanks are weakly concave, with a marked flexure. The long neck is cylindrical and occupies one half (50%) of the total length. The aperture flares, and is surrounded by a slightly denticulated collarette. The vesicle surface is smooth, with some isolated small tubercules (less than 2 μm in height). No basal features are discernible, as the base is flattened inwards in the specimen.

**Remarks**: The single specimen assigned to *Sphaerochitina ricardi* Díez & Cramer, 1978 [32] herein bears a similar vesicle shape and neck proportion to the original description of the species. Díez & Cramer [32] mentioned surface ornamentation composed of small granules or spines widely spaced which is similar to the isolated small tubercles of the Tacobo specimen. However, our specimen is slightly larger than previous records of this species, except those of Winchester-Seeto & Carey [34], assigned to *Angochitina* cf. *Sphaerochitina ricardi* which have a total length similar to that of the Los Monos Formation. We agree that those specimens of *Angochitina* cf. *Sphaerochitina ricardi* indeed are not assignable to *S*. *ricardi*.

**Occurrence:** Global distribution: *Sphaerochitina ricardi* was recorded in Iberia (Armorica terrane) from the Emsian to early Eifelian of La Vid Formation by Díez & Cramer [32], and the early Givetian of Naranco, Huergas and Gustalapiedra formations by Askew & Russell [33]. In Northern Gondwana, Winchester-Seeto & Carey [34] recorded therein a ‘cf.’ species in the Pragian of the Point Hibbs Formation, SW Tasmania.

*Sphaerochitina ricardi* Díez & Cramer, 1978 is recorded herein from the early Givetian in the TCB X-1001-Tacobo borehole.

Family **Lagenochitinidae** Eisenack, 1931 [25] emend. Paris, 1981 [27]

Subfamily **Lagenochitininae** Paris, 1981 [27]

Genus ***Lagenochitina*** Eisenack, 1931 [25] emend. Paris et al., 1999 [23]

Type species: *Lagenochitina baltica* Eisenack, 1931 [25]

*Lagenochitina amottensis* Grignani & Mantovani, 1964 [35]

.1964 *Lagenochitina amottensis*; Grignani & Mantoviani, p. 247, pl. 2, figs 18–19. [35]

.2013 *Lagenochitina amottensis*; Zhang et al., p. 2. [36]

Fig 4E–4G.

**Type specimen:** Holotype: Grignani & Mantoviani [35 pl. 2, fig. 18].

**Material**: Three well-preserved specimens and one assigned with uncertainty. In total, four specimens were observed and measured from sample 9162 (4975 m) (Table 3).

**Table 3:** *Lagenochitina amottensis* measurements.

Dimensions (μm)	L	ln	lc	D	dn	da	%neck	fracc	L:D	lc:ln	D:dn	L:lc
Mean	176	77	96	40	40	41	45%	4/9	2:1	1:1	2:1	2:1
Min	132	64	68	31	36	31						
Max	209	100	123	53	47	53						

**Description**: The vesicle chamber is ovoid, with a very weak to inconspicuous basal margin and a convex-rounded base. The flanks are convex, and display a distinct flexure with a gentle shoulder. The neck occupies less than a half of the total length (45%), is cylindrical, and on some occasions may be slightly flared towards the aperture. The vesicle wall is smooth, without any ornamentation. The bases of all specimens are flattened inwards, and as such basal features cannot be discerned.

**Remarks**: The Tacobo borehole specimens strongly resemble *Lagenochitina amottensis* Grignani & Mantovani, 1964 [35], in vesicle shape and measurements. Although the original description of the species is based on light microscope images, the similar outline and description allow for the confident assignation of the species. The only difference is the length of the neck which was recorded in the type material as being half of the total length, but in our specimens reach a maximum of 45% (4/9) occupied by the neck.

**Occurrence:** Global distribution: *Lagenochitina amottensis* was recorded in Northern Gondwana from the Middle and Upper Devonian of the OUM DOUL 1 borehole, Morocco [35], and Givetian of the Jinbo Formation, South China [36]. There are no previous records from Euramerica or Western Gondwana.

*Lagenochitina amottensis* Grignani & Mantovani, 1964 [35], is recorded herein from the early Givetian of the TCB X-1001-Tacobo borehole.

*Lagenochitina sommeri* (Lange, 1952) [37]

.1952 *Desmochitina sommeri*; Lange, pp. 379–382, pl. 19, figs 13–22. [37]

.1996 *Lagenochitina sommeri*; Ottone, p. 143, pl. 11, fig. 6. [30]

.2002 *Desmochitina*? *sommeri*; Grahn & Melo, p. 137. [38]

.2005 *Lagenochitina sommeri*; Grahn & Melo, p. 19, pl. 2, fig. 4; pl. 4, fig. 1. [39]

Fig 4H.

**Type specimen:** Holotype: Lange [37 pl. 19, fig. 13].

**Material**: One complete and well-preserved specimen was observed and measured from sample 9164 (5065 m) (Table 4).

**Table 4:** *Lagenochitina sommeri* measurements.

Dimensions (μm)	L	ln	lc	D	dn	da	%neck	fracc	L:D	lc:ln	D:dn	L:lc
Mean	128	41	86	74	33	43	30%	1/3	2:1	2:1	2:1	1:1

**Description**: The vesicle chamber is ovoid, with a broadly rounded to inconspicuous basal margin and a convex base. The flanks are convex, with a gentle flexure and weakly developed shoulders. The cylindrical neck occupies approximately one-third (30%) of the total length, terminating in a flaring collarette. The surface of the vesicle is predominantly smooth, although some granules may occur. Basal features are not discernible, due to inward flattening of the base of the specimen.

**Remarks**: The specimen assigned to *Lagenochitina sommeri* (Lange, 1952) [37], herein matches in dimensions and general outline with the type material. Lange placed this species within the genus *Desmochitina*, though it was later assigned by Ottone [30] and Grahn and Melo [38,39] to *Lagenochitina*, due to the clear differentiation of the neck and body and glabrous surface.

**Occurrence:** Global distribution: *Lagenochitina sommeri* is restricted to Western Gondwana. Brazil: Middle Devonian of the lower Barreirinha Formation, Amazonas Basin [37]; early Frasnian [38]; Frasnian of the upper Pimenteira Formation, Parnaíba Basin [39]. Northwestern Argentina: Devonian of the Los Monos Formation, Quebrada Galarza well [30].

*Lagenochitina sommeri* (Lange, 1952) [37] is recorded herein from the early Givetian in the TCB X-1001-Tacobo borehole.

*Lagenochitina tacobensis* sp. nov.

Fig 4I–4N.

**Derivation of the name:** After the TCB X-1001-Tacobo borehole, southern Bolivia, from which the type material is described.

**Type specimen:** Holotype: Fig 4N

**Material**: Sixteen specimens were observed and measured from samples 9160 (4830 m), 9161 (4870 m), 9162 (4975 m), 9163 (5035 m) and 9164 (5065 m) (Table 5).

**Table 5:** *Lagenochitina tacobensis* measurements.

Dimensions (μm)	L	ln	lc	D	dn	da	%neck	fracc	L:D	lc:ln	D:dn	L:lc
Mean	203	54	149	73	39	38	30%	1/4	3:1	3:1	2:1	1:1
Min	130	32	85	34	19	22						
Max	254	81	205	91	54	52						

**Diagnosis**: *Lagenochitina* species with an ovoid elongated vesicle shape with a short neck occupying one-quarter of the total length and smooth surface.

**Description**: The vesicle chamber is ovoid elongated, with a convex base and well-rounded basal margin. The flanks are generally convex but may seem to be straight in some specimens. The flexure is weak and the shoulder is inconspicuous to absent. The cylindrical neck covers approximately one-quarter (30%) of the total length, and the aperture bears a non-flaring slightly denticulate collarette. The vesicle wall is smooth with some isolated tubercules. No mucron or basal features are observed.

**Remarks**: *Lagenochitina tacobensis* sp. nov. differs from *Lagenochitina claviformis* Rauscher & Doubinger, 1967 [40] in that the latter has a clavate vesicle chamber and longer total length. *Lagenochitina cylindrica* Eisenack, 1931 [25] is larger than *L*. *tacobensis* sp. nov. and the vesicle shape is cylindrical. *Lagenochitina longiformis* Obut, 1995 [41], has a similar outline, but the neck is much longer than our species and the overall size is larger. *Lagenochitina pirum* Achab, 1982 [42] has a conical vesicle shape.

**Occurrence:** *Lagenochitina tacobensis* sp. nov. is recorded from the Tacobo X-1001 borehole in southern Bolivia, assigned to the early Givetian. The holotype is recorded in sample 9161 (4870 m).

*Lagenochitina vitrea* (Taugourdeau, 1962) [43]

.1962 *Sphaerochitina vitrea*; Taugourdeau, pl. 1, figs 16–17. [43]

.1964 *Sphaerochitina vitrea*; Cramer, p. 353, pl. XXI, figs. 10–13. [44]

.1967 *Lagenochitina vitrea*; Cramer, pp. 103–104, pl. II, fig. 47; pl. III, figs 67, 73, 80–81, 86. [45]

Fig 4O and 4P.

**Type specimen**: Holotype: Taugourdeau [43, pl. 1, fig. 16].

**Material**: Two moderately well-preserved and six broken and damaged specimens were observed and measured from samples 9162 (4975 m) and 9164 (5065 m) (Table 6).

**Table 6:** *Lagenochitina vitrea* measurements.

Dimensions (μm)	L	ln	lc	D	dn	da	%neck	fracc	L:D	lc:ln	D:dn	L:lc
Mean	211	101	110	92	39	45	50%	1/2	2:1	1:1	2:1	2:1
Min	133	68	65	78	29	30						
Max	280	135	146	111	51	61						

**Description**: The vesicle chamber is cylindroconical to subconical, with a slightly convex base and well-rounded basal margin. The flanks are straight to slightly convex, and display a subtle flexure with a weakly developed shoulder. The cylindrical neck covers approximately one-half (50%) of the total length, and flares slightly towards the aperture. The vesicle wall is smooth with some isolated tubercules. No mucron or other basal features are observed.

**Remarks**: Taugourdeau [43] described and illustrated this species with only light microscope images. He describes the specimens as completely transparent, indicating that it is a diagnostic characteristic. However, it is a preservational feature which cannot be considered as diagnostic. The vesicle outline and measurements are similar to the type material. The length of the neck of the Tacobo specimens is half of the total length, thus slightly shorter than that of the type material.

**Occurrence:** Global distribution: *Lagenochitina vitrea* was recorded in Northern Gondwana from the middle and upper Llandovery strata of wells in the Sahara [43]. In Iberia (Armorica terrane) was recorded from the upper part of the Formigoso Formation and the lower part of the San Pedro Formation, both of Silurian age [44,45]. There are no previous records from Western Gonwana.

*Lagenochitina vitrea* (Taugourdeau, 1962) [43] is recorded herein from the early Givetian in the TCB X-1001-Tacobo borehole.

*Lagenochitina* cf. *pirum* (Achab, 1982) [42]

1982 *Conochitina pirum*; Achab, pp.1298–1302, pl. 3, figs1–12. [42]

1995 *Conochitina pirum*; Achab & Asselin, pl. I, fig. 12. [46]

1995 *Conochitina pirum*; Wang & Chen, pl.2, figs 1, 10, 13; pl. 3, fig. 1. [47]

1996 *Conochitina pirum*; Chen et al., pl.1, fig. 10. [48]

2001 *Lagenochitina pirum*; Albani et al., pl. 3, figs 5–9. [49]

2007 *Lagenochitina praepirum*; Tang et al., p. 96, pl.2, figs 1–6, 9, 10. [50]

2009 *Lagenochitina pirum*; Chen et al., p. 324, pl. II, figs 7, 12–13. [51]

Figs 4Q–4S, 6A and 6B.

**Material**: Twenty-two specimens were observed and measured from samples 9161 (4870 m), 9162 (4975 m), 9163 (5035 m), 9164 (5065 m) and 1966 (5170 m) (Table 7).

**Table 7:** *Lagenochitina* cf. *pirum* measurements.

Dimensions (μm)	L	Ln	lc	D	dn	da	%neck	fracc	L:D	lc:ln	D:dn	L:lc
Mean	167	42	125	80	42	42	25%	1/4	2:1	3:1	2:1	1:1
Min	124	21	90	63	31	31						
Max	215	73	167	95	74	58						

**Description**: The vesicle chamber is subconical to subcylindrical, with a convex base and distinctly rounded basal margin. The flanks are straight to weakly concave, and display an inconspicuous to subtle flexure. The shoulder is generally absent, but on some specimens a subtle inflexion is apparent. The short neck is cylindrical, and forms approximately one-quarter (25%) of the total length. The aperture is bordered by a slightly flaring collarette, which is simple to gently denticulated. The vesicle surface is smooth to slightly granulate. A mucron is observed at the centre of the base on some specimens, as the only feature present at the base.

**Remarks**: Although the Tacobo specimens are similar to *Lagenochitina pirum* (Achab, 1982) [42] in the general vesicle outline, they are smaller in size and with a smooth vesicle surface instead of rugose. This species was previously recorded only from the Early and Middle Ordovician. Due to the large time interval between the two records (Ordovician and Devonian), the Tacobo specimens could represent a new species, so they are doubtfully assigned to *Lagenochitina pirum*.

**Occurrence**: Global distribution: *Lagenochitina pirum* (Achab, 1982) [42] has been described from the Early Ordovician in Euramerica by Achab and Achab and Asselin [42,46,52,53], and from the middle Ordovician in Eastern Gondwana by Chen et al. and Tang et al. [48,50,51]. There are no previous records of this species in the Silurian or the Devonian.

*Lagenochitina* cf. *pirum* (Achab, 1982) [42] is recorded herein from the early Givetian in the TCB X-1001-Tacobo borehole.

*Lagenochitina* sp. A.

Fig 5C.

**Fig 5:**
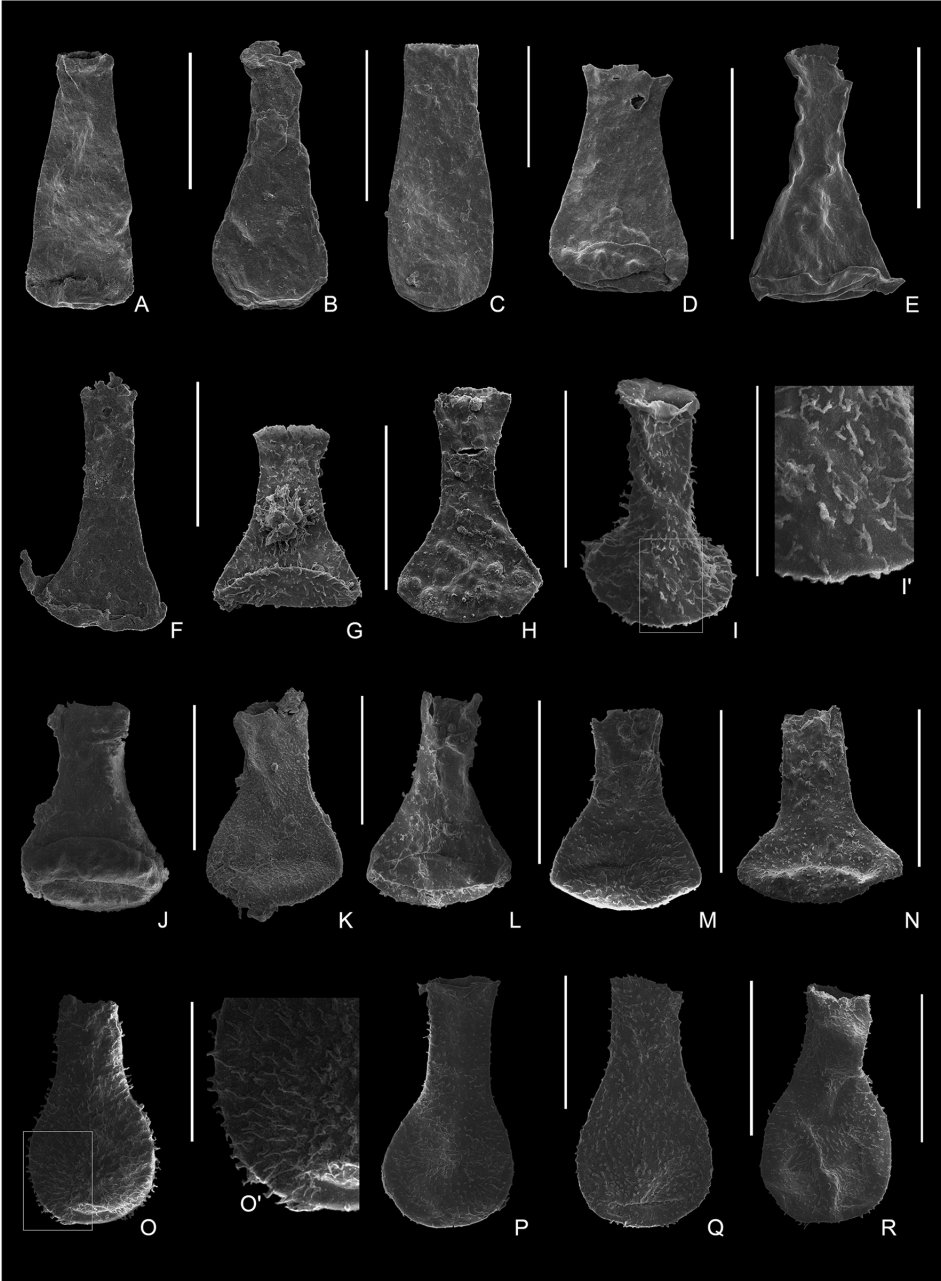
Plate 2. (A–B) *Lagenochitina* cf. *pirum* (Achab, 1982), (A) 9162-S11-55, from Sample 9162; (B) 9164-S17-50, from Sample 9164. (C) *Lagenochitina* sp. A, 9162-S12-06, from Sample 9162. (D) *Lagenochitina* sp B, 9162-S14-47, from Sample 9162. (E–F) *Cyathochitina* cf. *campanulis* Boneham & Masters, 1973, (E) 9160-S24-36, from Sample 9160; (F) 9163-S22-98, from Sample 9163. (G–N) *Fungochitina pilosa* (Collinson & Scott, 1958), (G–I) 9158-S8-03, 9158-S8-04, 9158-S21-47, from Sample 9158; (I’) detail of the ornamentation showing randomly distributed simple spines. (J–L) 9161-S19-49, 9161-S19-56, 9161-S21-20, from Sample 9161; (M) 9162-S15-20, from Sample 9162; (N) 9164-S18-09, from Sample 9164. (O–R) *Angochitina galarzae* Ottone, 1996, (O–P) 9158-S21-36, 9158-S21-40, from Sample 9158; (O’) detail of the ornamentation showing simple long hairs or spines. (Q–R) 9161-S20-11, 9161-S20-31, from Sample 9161. All scale bars represent 100 μm.

**Material**: One well-preserved and three fractured specimens were observed and measured from samples 9161 (4870 m) and 9162 (4975 m) (Table 8).

**Table 8:** *Lagenochitina* sp. A. measurements.

Dimensions (μm)	L	ln	lc	D	dn	da	%neck	fracc	L:D	lc:ln	D:dn	L:lc
Mean	212	48	164	81	55	54	25%	1/4	3:1	3:1	1:1	1:1
Min	207	42	126	76	51	49						
Max	218	54	175	87	60	61						

**Diagnosis**: *Lagenochitina* species with a wide neck almost equal to the diameter of the chamber (Dp:dn = 1).

**Description**: The vesicle chamber is elongated ovoid to subcylindrical, with a convex base and rounded basal margin. The flanks are straight to slightly convex, with a subtle flexure. The neck is short and wide, occupying approximately one-quarter (25%) of the total length. Its shape is cylindrical and non-flaring at the aperture. The vesicle surface is smooth to weakly granulated. No basal features are discernible, as a consequence of inward flattening of the base.

**Remarks**: Although the genus *Euconochitina* is also recognized by the gentle flexure and lack of a shoulder, the present specimens are assigned to *Lagenochitina* due to the elongated ovoid vesicle shape, differentiated neck and body chamber, and smooth surface. It differs from other species of *Lagenochitina* by the width of the neck which is almost equal to the diameter of the chamber. The specimens recovered are scarce, completely flattened and mostly fractured, and as such, they are retained in open nomenclature.

**Occurrence**: *Lagenochitina* sp. A is recorded herein from the early Givetian in the TCB X-1001-Tacobo borehole.

*Lagenochitina* sp. B.

Fig 5D.

**Material**: One moderately well-preserved specimen was observed and measured from sample 9162 (4975 m) (Table 9).

**Table 9:** *Lagenochitina* sp. B. measurements.

Dimensions (μm)	L	ln	lc	D	dn	da	%neck	fracc	L:D	lc:ln	D:dn	L:lc
Mean	133	29	104	81	44	54	20%	1/5	4:1	2:1	2:1	1:1

**Diagnosis**: *Lagenochitina* species with a conical chamber, flat base and short neck.

**Description**: The vesicle chamber is conical, with a flat base and broadly rounded basal margin. The flanks are straight and display no distinct flexure. The neck is short, occupying approximately one-fifth (20%) of the total length, with a gently flaring denticulated collarette. The vesicle surface is generally smooth, with some isolated granules. No basal features are discernible, as a consequence of inward flattening of the base.

**Remarks**: The specimen assigned to this genus displays the main characteristics of *Lagenochitina*, but due to the paucity of material and lack of distinguishing features for identification at species level, it is retained in open nomenclature.

**Occurrence**: *Lagenochitina* sp. B is recorded herein from the early Givetian in the TCB X-1001-Tacobo borehole.

Subfamily **Cyathochitininae** Paris, 1981 [27]

Genus ***Cyathochitina*** Eisenack, 1955 [31]

Type species: *Conochitina campanulaeformis* Eisenack, 1931 [25]

*Cyathochitina* cf. *campanulis* Boneham & Masters, 1973 [54]

1973 *Cyathochitina campanulis*; Boneham & Masters, p.94, figs 8–9. [54]

Fig 5E and 5F.

**Type specimen:** Holotype: Boneham & Masters [54 fig. 9].

**Material**: Two moderately well-preserved specimens were observed and measured from samples 9160 (4830 m) and 9163 (5035 m) (Table 10).

**Table 10:** *Cyathochitina* cf. *campanulis* measurements.

Dimensions (μm)	L	ln	lc	D	dn	da	%neck	fracc	L:D	lc:ln	D:dn	L:lc
Mean	165	81	84	86	32	41	50%	1/2	2:1	1:1	3:1	2:1
Min	160	81	79	86	30	40						
Max	171	82	89	86	34	43						

**Description**: The vesicle chamber is conical, with a concave base inferred from its inward flattening. The flanks are straight to weakly concave with a gentle flexure. The long neck is cylindrical and occupies approximately one-half (50%) of the total length. The aperture is outlined by a flaring, slightly denticulated collarette. The surface of the vesicle is smooth with some occasional small tubercules (less than 2 μm in height). The base bears a single complete carina which is c. 13 μm wide. The carina seems to have been positioned normal to the longitudinal axis, however, due to flattening it appears at a lower angle.

**Remarks**: Our specimens strongly resemble *Cyathochitina campanulis* Boneham & Masters, 1973 [54], in both vesicle shape and dimensions. *C*. *campanulis* is morphologically very close to the more widely recorded species *C*. *campanulaeformis* (Eisenack, 1931) [25] and *C*. *kuckersiana* (Eisenack, 1934) [55] and probably related, as indicated by Boneham & Masters (1973) [54]. Even though the Tacobo specimens are scarce and flattened, strong similarities with *C*. *campanulis* are discernable. Nonetheless, awaiting further studies on intraspecific variation between these species, they are assigned to *C*. *campanulis* but with uncertainty.

**Occurrence:** Global distribution: *Cyathochitina campanulis* has only been recorded by Boneham & Masters [54] from Euramerica, in the Silurian Osgood Member of the Salamonie Dolomite (Indiana, USA).

*Cyathochitina* cf. *campanulis* Boneham & Masters, 1973 [54] is recorded herein from the early Givetian in the TCB X-1001-Tacobo borehole.

Subfamily **Angochitininae** Paris, 1981 [27]

Genus *Fungochitina* Taugourdeau, 1966 [56]

Type species: *Conochitina fungiformis* Eisenack, 1931 [25]

*Fungochitina pilosa* (Collinson & Scott, 1958) [57]

.1958 *Sphaerochitina pilosa*; Collinson & Scott, p. 21–22, pl. 3, figs 1–5. [57]

.1958 *Sphaerochitina schwalbi*; Collinson & Scott, p. 23, pl. 3, figs 6–10. [57]

.1965 *Sphaerochitina pilosa*; Taugourdeau, pp. 66–67, pl. 1, figs 28, 30. [58]

.1972 *Sphaerochitina pilosa*; Urban, p. 25, pl. 4, figs 1–3. [59]

.1973 *Sphaerochitina pilosa*; Urban & Newport, p. 241, pl. 2, figs 1–16. [60]

.1981 *Angochitina pilosa*; Paris, p. 60, pl. 3, figs 7–8, 11, 13–14, pl. 4, fig. 13. [27]

.1985 *Fungochitina pilosa*; Paris et al., p. 49, pl. 28, figs 3, 6, 10: a–b. [61]

.1988 *Fungochitina pilosa*; Boumendjel et al., p. 341, pl. 5, fig. 9. [62]

.1996 *Fungochitina pilosa*; Paris, p. 544, pl. 3, fig. 5. [63]

.2002 *Fungochitina pilosa*; Grahn et al., p. 139, pl. 8, figs G–H. [64]

.2004 *Fungochitina pilosa*; Grahn & Melo, p. 73, pl. 1, figs 6–7. [65]

.2011 *Fungochitina pilosa*; Noetinger & di Pasquo, p. 207, figs 6: N, appendix figs 7: F–H, 8: E–F. [66]

Fig 5G–5N.

**Type specimen:** Holotype: Collinson & Scott [57 pl. 3, fig. 2].

**Material**: Twenty-three well-preserved specimens, and two assigned with some uncertainty, were observed and measured from samples 9158 (4725 m), 9160 (4830 m), 9161 (4870 m), 9162 (4975 m), 9163 (5035 m) and 9164 (5065 m) (Table 11).

**Table 11:** *Fungochitina pilosa* measurements.

Dimensions (μm)	L	ln	lc	D	dn	da	%neck	fracc	L:D	lc:ln	D:dn	L:lc
Mean	145	62	83	91	40	45	40%	2/5	2:1	1:1	2:1	2:1
Mín	106	43	44	76	30	32						
Máx	190	83	113	107	52	62						

**Fig 6:**
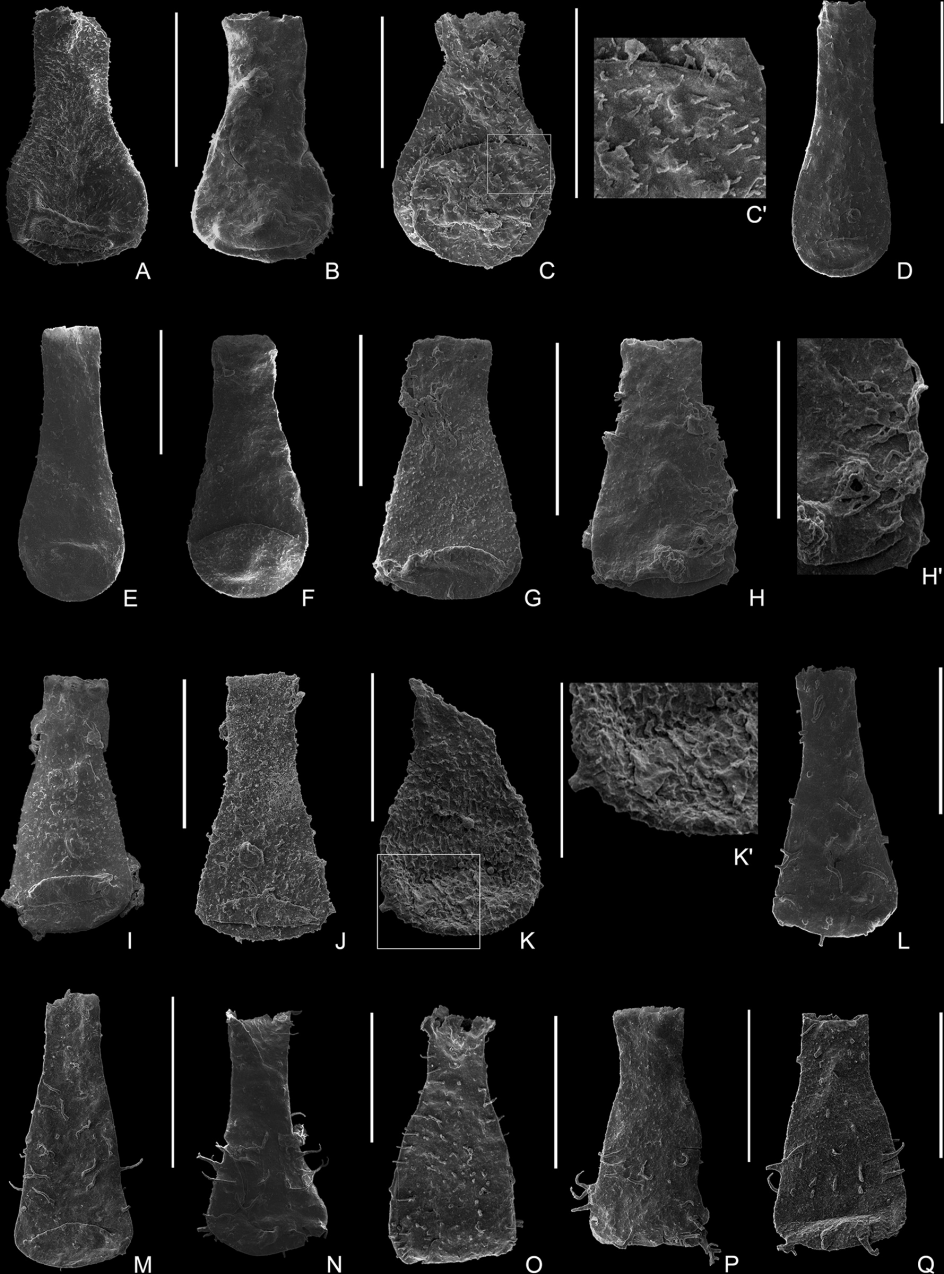
Plate 3. (A–B) *Angochitina galarzae* Ottone, 1996, 9161-S20-53, 9161-S19-37, from Sample 9161. (C) *Angochitina* aff. *capillata* Eisenack, 1937, 9162-S14-91, from Sample 9162; (C’) detail of the ornamentation showing simple hairs or spines densely and randomly distributed. (D–E) *Angochitina* cf. *elongata* Eisenack, 1931, 9161-S19-20, 9161-S20-61, from Sample 9161. (F) *Angochitina* sp., 9161-S20-48, from Sample 9161. (G–J) *Muscochitina*? sp. A, 9162-S15-71, 9162-S15-46, 9162-S7-35, 9162-S7-04, from Sample 9162; (H’) detail of the ornamentation showing a stout net partially covering the vesicle surface. (K) *Muscochitina*? sp. B, 9163-S22-30, from Sample 9163; (K’) detail of the vesicle ornamentation and the two types of processes at the base. (L–N) *Ramochitina autasmirimense* Grahn & Melo, 2004, (L–M) 9162-S15-11, 9162-S17-11, from Sample 9162, (N) 9161-S20-78, from Sample 9161. (O–Q) *Ramochitina* cf. *autasmirimense* Grahn & Melo, 2004, (O) 9158-S8-05, from Sample 9158; (P–Q) 9163-S22-03, 9163-S22-92, from Sample 9163. All scale bars represent 100 μm.

**Description**: The vesicle chamber is conical to sub-ovoid, with a slightly convex base and clearly rounded basal margin. The flanks are straight to weakly convex, with a gentle flexure that in some occasions is marked. The shoulder is weakly developed to absent. The cylindrical neck occupies approximately two-fifths (40%) of the total length and flares towards the simple aperture. The surface of the vesicle is covered with randomly distributed simple small spines up to 3 μm over the neck, that increase in size towards the base of the vesicle reaching up to 18 μm in length (Fig 6I’). In some specimens, the surface is highly eroded and broken spines may look like small tubercles—when a detailed observation is made, however, the scars and broken spines can be recognized clearly. No mucron is present.

**Remarks**: The specimens assigned to *Fungochitina pilosa* herein strongly resemble those originally described by Collinson & Scott [57] in both vesicle shape and measurements. Some specimens display areas denuded of spines and which appear to be smooth, although attachment scars can be recognized. The spiny ornamentation normally decreases in size from the base to the neck until reaching the size and shape of granules.

This species was originally considered as belonging to the genus *Sphaerochitina*, but was reassigned to *Fungochitina* by Paris [27] based on its conical, almost “fungic”, vesicle shape and randomly distributed spines. *Sphaerochitina schwalbi* Collinson & Scott, 1958 [57], is very similar to *F*. *pilosa*, but is differentiated by a slightly different vesicle shape and size. These are features that allow for a clear separation of both species, and therefore we consider *S*. *schwalbi* a junior synonym of *F*. *pilosa*. To further compound matters, *F*. *pilosa* is considered to be a wastebasket for some authors, but the strong similarities of our specimens to the original description of the species allows for the confident designation of the Tacobo specimens herein to *F*. *pilosa*.

**Occurrence:** Global distribution: *Fungochitina pilosa* was recorded in Euramerica from the Middle Devonian Cedar Valley Formation and Wapsipinicon Formation, USA [57,59,60] and in the Middle Frasnian from Boulonnais, USA [58]. Northern Gondwana: recorded from the Givetian of the Méderba and Alrar formations, southeastern Algeria [62]. Western Gondwana: from the Middle-Late Devonian of the São Domingos and Lima formations, Paraná Basin of Brazill and Paraguay [64]; from the Middle Devonian of the Ereré Formation, Amazonas Basin, Brazil [65]; from Devonian of the San Antonio X-1 borehole, Tarija Basin, Argentina [66].

*Fungochitina pilosa* Collinson & Scott, 1958) is recorded herein from the early and middle Givetian in the TCB X-1001-Tacobo borehole.

Genus ***Angochitina*** Eisenack, 1931 [25]

Type species: *Angochitina echinata* Eisenack, 1931 [25]

*Angochitina galarzae* Ottone, 1996 [30]

.1996 *Angochitina galarzae*; Ottone, p. 142, pl. 11, fig. 10, pl. 12, figs 3, 5–6. [30]

**Fig 7:**
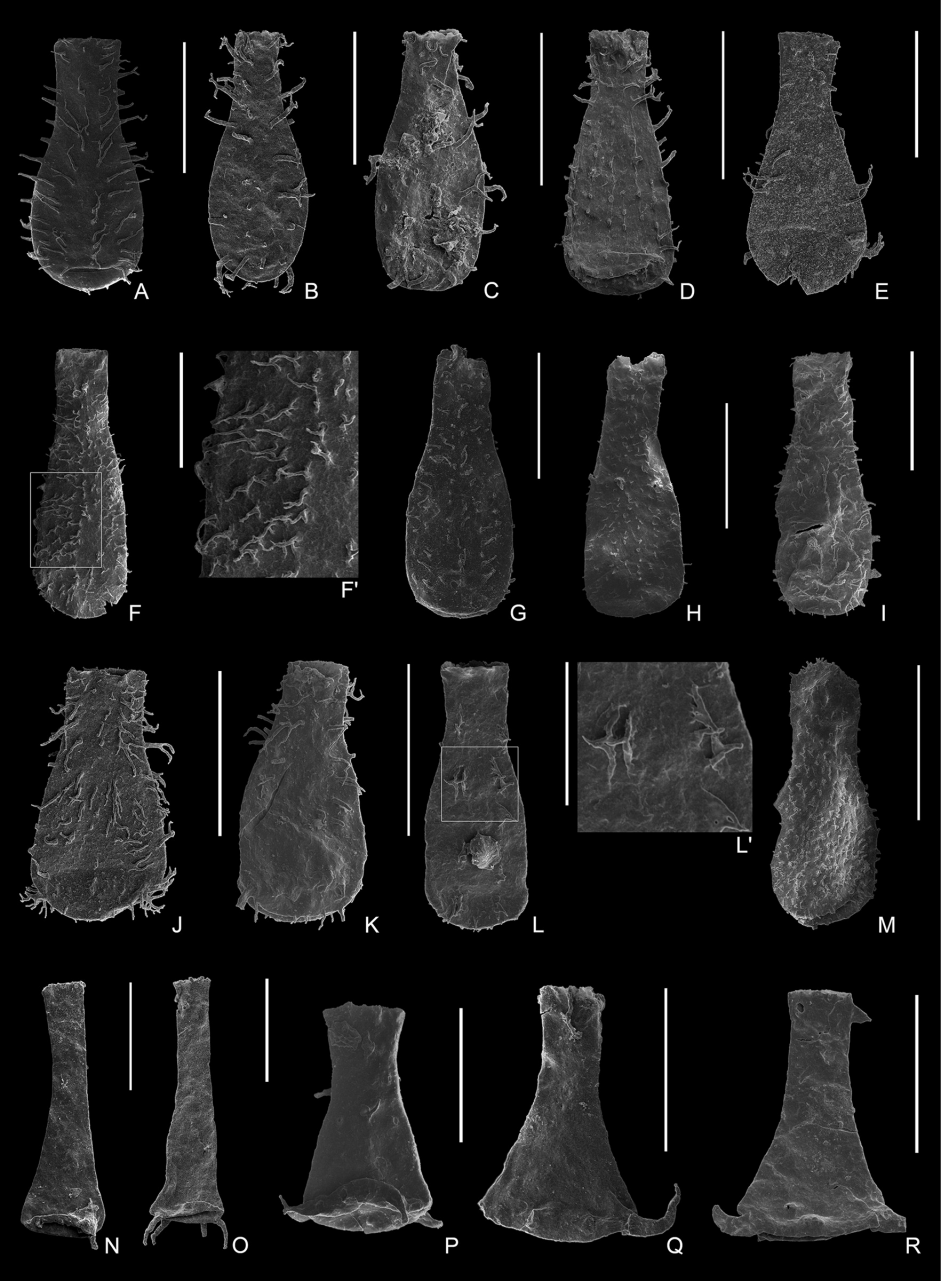
Plate 4. (A–E) *Ramochitina boliviensis* Grahn, 2002, (A) 9161-S19-18, from Sample 9161; (B–C) 9163-S22-90, 9163-S22-01, from Sample 9163; (D–E) 9162-S7-23, 9162-S7-07, from Sample 9162. (F–I) *Ramochitina candelariaensis* sp. nov., (F) 9160-S24-17, holotype, from Sample 9160 (F’) detail of the ornamentation showing densely aligned simple, lambda or multirooted spines; (G–I) 9161-S20-73, 9161-S20-04, 9161-S20-56, from Sample 9161. (J–K) *Ramochitina stiphrospinata* Grahn & Melo, 2005, (J) 9163-S22-19, from Sample 9163; (K) 9164-S18-23, from Sample 9164. (L) *Ramochitina* sp. cf. *durandii* n. n. Pérez-Leytón, 2007, 9160-S24-14, from Sample 9160; (L’) detail of the ornamentation showing birooted, multirooted and coalescent spines aligned. (M) *Ramochitina* sp.; 9161-S21-16, from Sample 9161. (N–O) *Ancyrochitina biconstricta* (Lange, 1949), 9164-S17-27, 9164-S17-56, from Sample 9164. (P–R) *Ancyrochitina cornigera* Collinson & Scott, 1958, (P) 9158-S21-35, from Sample 9158; (Q) 9164-S17-43, from Sample 9164; (R) 9162-S12-31, from Sample 9162. All scale bars represent 100 μm.

.2011 *Angochitina galarzae*; Noetinger & di Pasquo, p. 210, pl. appendix fig. 6: M, fig. 7: C. [66]

.2015 *Angochitina galarzae*; di Pasquo et al., p. 79, fig. 8: 10. [67]

Figs 5O–5R, 6A and 6B.

**Type specimen**: Holotype: Ottone [30 pl. 11, fig. 10].

**Material**: Nine well-preserved specimens, plus two assigned with a degree of uncertainty, were observed and measured from samples 9158 (4725 m), 9160 (4830), 9161 (4870 m), 9162 (4975 m), 9163 (5035 m) and 9164 (5065) (Table 12).

**Table 12:** *Angochitina galarzae* measurements.

Dimensions (μm)	L	ln	lc	D	dn	da	%neck	fracc	L:D	lc:n	D:dn	L:lc
Mean	163	69	94	87	45	45	40%	2/5	2:1	1:1	2:1	2:1
Min	133	46	85	71	37	36						
Max	188	91	102	98	52	58						

**Description**: The vesicle chamber is ovoid, with an inconspicuous basal margin and convex base, although in some specimens due to inward flattening the base seems to be concave. The flanks are clearly convex, with marked flexure and a very weak to inconspicuous shoulder. The neck is cylindrical and displays mostly non-flaring slightly denticulate collarette, although in some instances the collarette can be weakly flaring. The ornamentation over the vesicle wall comprises simple long hairs or spines (from 2 to 10 μm long, and 0.5 to 2 μm in diameter) with even distribution over the entire surface (Fig 5O’). The spacing between spines is c. 1 to 5 μm. The base bears the same ornamentation as the rest of the vesicle.

**Remarks**: Specimens from the Tacobo borehole that are assigned confidently to *Angochitina galarzae* resemble the original description of Ottone [30] in terms of vesicle shape, dimensions and ornamentation. The specimens assigned uncertainly display a slightly longer neck, reaching almost one-half of the total length and a slightly flaring collarette which is not observable in the type material. Nevertheless, Ottone [30] illustrated some variation in the length of the neck of his specimens, suggesting that this feature may vary from specimen to specimen. Another difference is the gentle flaring collarette at the end of the aperture discernable in some specimens. This is a subtle variation and it may be caused by the flattening of the material, as some fractures can be discernible at the aperture of these specimens. These two slightly different characteristics are not distinct enough to allow for splitting of the taxon, hence all specimens are assigned herein to *Angochitina galarzae*.

**Occurrence**: Global distribution: *Angochitina galarzae* is restricted to Western Gondwana. Northwestern Argentina: late Givetian–early Frasnian of the Los Monos Formation and the Givetian of the San Antonio x-1 Borehole, Tarija Basin [30,66]. South Bolivia: Givetian of the Los Monos Formation [67].

*Angochitina galarzae* Ottone, 1996 [30], is recorded herein from the early and middle Givetian in the TCB X-1001-Tacobo borehole.

*Angochitina* aff. *capillata* Eisenack, 1937 [68]

1937 *Angochitina capillata*; Eisenack, p. 225, pl. 15, fig. 13. [68]

1973 *Angochitina capillata*; Urban & Newport, p. 240, pl. 1, figs 9, 13. [60]

2019 *Angochitina capillata*; Askew & Russell, p. 76, pl. I, fig. 5. [33]

Fig 6C.

**Type specimen**: Holotype: Eisenack [68, pl. 15, fig. 13].

**Material**: One well-preserved specimen was observed and measured from sample 9162 (4975 m) (Table 13).

**Table 13:** *Angochitina* aff. *capillata* measurements.

Dimensions (μm)	L	ln	lc	D	dn	da	%neck	fracc	L:D	lc:ln	D:dn	L:lc
Mean	134	28	105	84	39	48	20%	1/5	2:1	4:1	2:1	1:1

**Description**: The vesicle chamber is ovoid, with a well-rounded basal margin and convex base. The flanks are convex, and a gentle flexure with an inconspicuous shoulder occurs. The neck is short, covering approximately one-fifth (20%) of the total length and is fringed by a simple thin-walled collarette at the aperture. The vesicle surface is covered by simple hairs or spines (length of the spines 3 to 5 μm and diameter 0.5 to 1 μm), which are densely and randomly distributed (space between spines c. 2 to 4 μm) (Fig 6C’). No basal features could be recognized as a consequence of inward flattening.

**Remarks**: *Angochitina capillata* Eisenack, 1937 [68] is a well-recorded species from the Ordovician and Silurian, but they differ from the Tacobo Middle Devonian specimens by several characteristics. The total length is similar in the entire specimen, although the measurement ratios are quite different. The neck in the Devonian specimens is significantly shorter, occupying one-fifth (20%) of the total length (Lp: Ln = 4:1), while in the older species this ratio normally is Lp: Ln = 2:1. The vesicle chamber from the Devonian specimens is mainly ovoid-spherical, as the older species is mainly ovoid elongated to subconical on some occasions. These two features are consistent and evident in both previous records of *Angochitina capillata*, and also in the Tacobo material. As the overall shape and morphological ratios are very different from older *Angochitina capillata* specimens, Middle Devonian species may well prove to be a separate taxon. Only one specimen from our material displays these noticeable characteristics, however, more material would be necessary to confirm a differentiation into two separate species–as such, it is retained in open nomenclature herein.

**Occurrence:** Global distribution: *Angochitina capillata* Eisenack, 1937 [68] has been extensively reported from Middle Ordovician to Devonian strata on all palaeocontinents. Although the Middle Devonian records are scarce, in Euramerica Urban & Newport [60] recorded the species from the Wapsipinicon Formation (Iowa, USA), and in Iberia (Armorica terrane) Askew & Russell [33] recorded it from the early Givetian of the Naranco, Huergas and Gustalapiedra formations.

*Angochitina* aff. *capillata* Eisenack, 1937 [68] is recorded herein from the early Givetian in the TCB X-1001-Tacobo borehole.

*Angochitina* cf. *elongata* Eisenack, 1931 [25]

1931 *Angochitina elongata*; Eisenack, p. 82, pl. 1, figs 8–9. [68]

1974 *Angochitina elongata*; Laufeld, p. 53, fig. 18. [69]

2007 *Angochitina elongata*; Nestor, p. 123, pl. 11, fig. P. [70]

2012 *Angochitina elongata*; Nestor, p. 157, fig. 6C. [71]

Fig 6D and 6E.

**Type specimen**: Holotype: Eisenack [25 pl. 1, fig. 9D].

**Material**: Five well-preserved specimens, and one assigned with uncertainty, were observed and measured from samples 9161 (4870 m) and 9162 (4975 m) (Table 14).

**Table 14:** *Angochitina* cf. *elongata* measurements.

Dimensions (μm)	L	ln	lc	D	dn	da	%neck	fracc	L:D	lc:ln	D:dn	L:lc
Mean	246	104	142	83	45	47	40%	2/5	3:1	1:1	2:1	1:1
Min	222	89	123	67	40	44						
Max	280	115	166	92	52	51						

**Description**: The vesicle chamber is elongated ovoid, with an inconspicuous basal margin and convex base. The flanks are straight to slightly convex, with a gentle to inconspicuous flexure. The neck is cylindrical with a thin-walled collarette which is simple and subtly flaring, and occupies approximately two-fifths (40%) of the total length. The vesicle surface is covered with randomly distributed, simple hairs or spines (1 to 7 μm long, 0.5 to 2 μm diameter at the base) spaced between 3 and 8 μm apart. No basal features could be recognized as a consequence of inward flattening of the base.

**Remarks**: *Angochitina elongata* was described by Eisenack [25] as an *Angochitina* species with a slender and elongated chamber that is generally longer than the neck with very short spines. A neotype was described by Eisenack [72], with denser ornamentation, a slightly different vesicle shape, and being less elongate than in the original description. Laufeld [69] distinguished two slightly different populations in Gotland, which he interpreted as intraspecific variations.

*Angochitina* cf. *elongata* is very similar to *Angochitina elongata* Eisenack, 1931 [25] but the former differs in having shorter spines and tubercules that are sparsely arranged, and smaller in size. The Tacobo specimens herein also have a weaker flexure and no shoulder. These two characteristics make the transition between the chamber and the neck less evident and create a slightly different general outline to the type material. The specimens assigned to *Angochitina* cf. *elongata* herein are similar to specimens of *Angochitina elongata* described by Nestor [73] from the Ludlow part of the Kaugatuma GI borehole (Estonia).

**Occurrence:** Global distribution: There are no previous records of *Angochitina elongata* Eisenack, 1931 [25] in the Middle Devonian since it has been exclusively recorded worldwide from the Late Silurian.

*Angochitina* cf. *elongata* Eisenack, 1931 [25] is recorded herein from the early Givetian in the TCB X-1001-Tacobo borehole.

*Angochitina* sp.

Fig 6F.

**Material**: One specimen was observed and measured from sample 9161 (4870 m) (Table 15).

**Table 15:** *Angochitina* sp. measurements.

Dimensions (μm)	L	ln	lc	D	dn	da	%neck	fracc	L:D	lc:ln	D:dn	L:lc
Mean	174	44	130	82	41	40	25%	1/4	2:1	3:1	2:1	1:1

**Diagnosis**: *Angochitina* species with an elongated ovoid vesicle chamber, a short cylindrical neck and small spines randomly distributed all over the vesicle surface.

**Description**: The vesicle chamber is an elongated ovoid, with a convex base and inconspicuous basal margin. The flanks are straight to slightly concave, with a weak flexure. The neck is cylindrical and occupies approximately one-quarter (25%) of the total length, and the aperture is fringed by a non-flaring collarette. The ornamentation consists of simple spines that cover the entire vesicle surface with a length between 1 and 4 μm, a diameter from 0.5 to 1 μm, and which are spaced approximately 1 to 4 μm apart. The specimen displays inward flattening at the base, and therefore no basal features can be discerned.

**Remarks**: The specimen assigned herein to *Angochitina* sp. resembles *Angochitina* cf. *elongata* in vesicle shape and size, but it has a much shorter neck and denser distribution of the spines. Other known *Angochitina* species have an elongated vesicle shape and long neck or have a short neck with an ovoid body. In the studied material there is only one specimen with these characteristics, therefore it is retained in open nomenclature.

**Occurrence**: *Angochitina* sp. is recorded herein from the early Givetian in the TCB X-1001-Tacobo borehole.

Genus ***Muscochitina*** Paris, 1981 [27]

Type species: *Muscochitina muscosa* Paris, 1981 [27]

*Muscochitina*? sp. A.

Fig 6G and 6J.

**Material**: Seven moderately well-preserved and three poorly-preserved specimens were observed and measured from samples 9162 (4975 m) and 9163 (5035 m) (Table 16).

**Table 16:** *Muscochitina*? sp. A. measurements.

Dimensions (μm)	L	ln	lc	D	dn	da	%neck	fracc	L:D	lc:ln	D:dn	L:lc
Mean	163	49	115	83	43	45	30%	1/3	2:1	2:1	2:1	1:1
Min	146	37	103	71	39	41						
Max	186	67	132	90	47	50						

**Diagnosis**: Angochitininae with a cylindroconical vesicle chamber, and the vesicle wall partially covered by a randomly distributed net.

**Description**: The vesicle chamber is cylindroconical to subovoid, with a broadly rounded basal margin. The flanks are straight to subtly convex, with a gentle flexure and inconspicuous to absent shoulder. The neck is cylindrical with an aperture that is slightly flaring and occupies approximately one-third (30%) of the total length. The ornamentation comprises a stout net that partially covers different parts of the specimen (Fig 6H’). In some specimens, it occurs at the basal margin or/and surrounding the neck, but it also may be randomly distributed over the vesicle. The spaces between the net are covered by simple and on some occasions multirooted and bifurcated spines. No basal features could be discerned as a consequence of inward flattening.

**Remarks**: The specimens assigned to *Muscochitina*? sp. A herein bears a unique ornamentation. The vesicle wall is partially covered by a stout net that on some occasions is aligned parallel to the longitudinal axis but also may be randomly distributed. The position of the ornamentation is also highly variable; it can be located only in one place or in multiple areas on the vesicle. It can occur over the neck, over the vesicle chamber or over the basal margin and any combination thereof.

A confident assignation of these specimens to the family Lagenochitinidae is due to a clear differentiation through the flexure of the neck and body. However, the genus assignation is doubtful due to the stout net distributed partially over different parts of the surface. The scarce and poorly preserved specimens of the Tacobo Borehole prevent the creation of a new genus.

**Occurrence**: *Muscochitina*? sp. A is recorded herein from the early Givetian in the TCB X-1001-Tacobo borehole.

*Muscochitina*? sp. B.

Fig 6K.

**Material**: One fractured specimen was observed and measured from sample 9163 (5035 m) (Table 17).

**Table 17:** *Muscochitina*? sp. B. measurements.

Dimensions (μm)	L	ln	lc	D	dn	da	%neck	fracc	L:D	lc:ln	D:dn	L:lc
Mean	145	44	102	90	48	-	30%	1/3	2:1	2:1	2:1	1:1

**Diagnosis**: Angochitininae with an ovoid vesicle chamber with the vesicle wall completely covered by a net.

**Description**: The vesicle chamber is ovoid, with a rounded basal margin and a flat to slightly convex base (inferred due to flattening). The flanks are convex and a gentle flexure with a slight shoulder occurs. The vesicle surface is covered by a stout net which is better developed near the base. The basal margin bears two different types of processes: the first is short (13 μm approximately), triangular, wide at the base (between 8 and 14 μm) and terminates sharply at the distal end. The second type, although broken, appears to be a thinner and tubular process (Fig 6K’).

**Remarks**: Only one specimen was assigned to *Muscochitina*? sp. B herein. Although the vesicle wall ornamentation is very similar to that of *Muscochitina*? sp. A, the net covers the entire surface, and is better developed near the base than over the neck. It also differs from *Muscochitina*? sp. A in the differentiated basal processes. The single specimen is provisionally assigned to *Muscochitina* based on the surface ornamentation.

**Occurrence**: *Muscochitina*? sp. B is recorded herein from the early Givetian in the TCB X-1001-Tacobo borehole.

Genus ***Ramochitina*** Sommer & van Boekel, 1964 emend. Paris et al., 1999 [74]

Type species: *Ramochitina ramosi* Sommer & van Boekel, 1964 [74]

*Ramochitina autasmirimense* Grahn & Melo, 2004 [65]

.2004 *Ramochitina autasmirimense*; Grahn & Melo, p.77, pl. 1, figs 8,9. [65]

.2015 *Ramochitina autasmirimense*; di Pasquo et al., p.77, fig. 8C. [67]

.2018 *Ramochitina autasmirimense*; Noetinger et al., p.103, pl. V, fig. 10. [75]

Fig 6L–6N.

**Type specimen**: Holotype: Grahn & Melo [65 pl. 1, fig. 8].

**Material**: Eight moderately well-preserved specimens were observed and measured from samples 9162 (4975 m) and 9164 (5065) (Table 18).

**Table 18:** *Ramochitina autasmirimense* measurements.

Dimensions (μm)	L	ln	Lc	D	dn	da	%neck	fracc	L:D	lc:ln	D:dn	L:lc
Mean	181	65	115	83	41	41	35%	1/3	2:1	2:1	2:1	2:1
Min	164	28	98	68	32	31						
Max	217	83	138	98	53	53						

**Description**: The vesicle chamber is conical to subconical, with a broadly rounded basal margin and a flat to slightly convex base. The flanks are straight and, on some occasions, slightly convex with a weak to absent flexure, and inconspicuous shoulder. The neck is cylindrical and occupies one-third (35%) of the total length. The aperture is fringed with a simple and non-flaring collarette. The vesicle surface is covered with simple and bifurcated spines which are aligned parallel to the longitudinal axis. The space between spines in a row is 5 to 10 μm, and their dimensions vary between 2 to 30 μm in length and 0.5 to 5 μm in width. No mucron or other basal features are present.

**Remarks**: Specimens assigned to *Ramochitina autasmirimense* Grahn & Melo, 2004 [65] herein bear the same diagnostic characteristics of the species. They only differ in having both simple and some bifurcated spines, which were not mentioned or described by the authors. However, Grahn and Melo [65 pl. 1, fig.8] illustrated one specimen that shows some bifurcated spines. With this characteristic being clearly developed in our material, we suggest that bifurcated spines, as well as simple spines, should be added to the diagnostic features of the species.

**Occurrence:** Global distribution: *Ramochitina autasmirimense* is restricted to Western Gondwana. Brazil: late Eifelian to early Givetian in the well 1-AM-1-AM and cores 21–23, Amazonas Basin [65], and Northern Argentina: from the Givetian and Frasnian of the Los Monos and Iquiri formations at Angosto del Pescado [67,75].

*Ramochitina autasmirimense* Grahn & Melo, 2004 [65], is recorded herein from the early and middle Givetian in the TCB X-1001-Tacobo borehole.

*Ramochitina* cf. *autasmirimense* Grahn & Melo, 2004 [65]

Fig 6O–6Q.

**Material**: Four moderately well-preserved specimens were observed and measured from samples 9158 (4725), 9162 (4975 m) and 9163 (5035) (Table 19).

**Table 19:** *Ramochitina* cf. *autasmirimense* measurements.

Dimensions (μm)	L	ln	lc	D	dn	da	%neck	fracc	L:D	lc:ln	D:dn	L:lc
Mean	162	47	114	82	40	46	30%	1/3	2:1	2:1	2:1	1:1
Min	157	41	105	74	35	45						
Max	165	55	124	91	47	47						

**Description**: The vesicle chamber is conical to subconical, with a rounded well-defined basal margin and a flat base. The flanks are generally straight, though on some occasions are slightly convex with a gentle flexure and shoulder. The neck is cylindrical and occupies one-third of the total length. The aperture is fringed with a denticulated collarette which may flare slightly. The vesicle surface is covered with simple and branched spines which are aligned parallel to the longitudinal axis. The space between spines in a row is 4 to 8 μm, and their dimensions vary between 6 to 25 μm in length and 0.6 to 6 μm in width. No mucron or other basal features are present.

**Remarks**: *Ramochitina* cf. *autasmirimense* is similar to *Ramochitina autasmirimense* Grahn & Melo, 2004 [65] in vesicle shape and size. It differs in having, occasionally, ornamentation of multi-branching spines and the presence of a gentle shoulder which is inconspicuous or absent in *R*. *autasmirimense*. Consequently, it is retained in open nomenclature.

**Occurrence**: *Ramochitina* cf. *autasmirimense* Grahn & Melo, 2004 [65] is recorded herein from the early and middle Givetian in the TCB X-1001-Tacobo borehole.

*Ramochitina boliviensis* Grahn, 2002 [7]

.2002 *Ramochitina boliviensis*; Grahn, p.323, figs 4: L, 5: E–F. [7]

.2002 *Ramochitina boliviensis*; Grahn et al., p.140, pl. 7, fig. I. [64]

.2015 *Ramochitina boliviensis*; di Pasquo et al., p.78, pl. 8, fig. 7. [67]

Fig 7A–7E.

**Type specimen:** Holotype: Grahn [7 fig. 5: F].

**Material**: Forty-six specimens; twenty-three well-preserved and twenty-three specimens assigned with some doubt, were observed and measured from samples 9160 (4830 m), 9161 (4870 m), 9162 (4975 m) and 9163 (5035 m) (Table 20).

**Table 20:** *Ramochitina boliviensis* measurements.

Dimensions (μm)	L	ln	lc	D	dn	da	%neck	fracc	L:D	lc:ln	D:dn	L:lc
Mean	184	53	132	76	39	39	30%	1/3	2:1	2:1	2:1	1:1
Min	154	24	100	58	24	28						
Max	230	97	167	97	48	52						

**Description**: The vesicle chamber is elongated ovoid, with a weak to inconspicuous basal margin and a convex base which, can be slightly concave in some instances. The flanks can be straight, but are mostly slightly convex. The flexure is weak to inconspicuous, and no shoulder is present. The neck is cylindrical and occupies one-third (30%) of the total length. The aperture is fringed with a simple and gently flaring collarette. The vesicle surface is covered with simple and bifurcated or multi-branching spines which are aligned parallel to the longitudinal axis. The space between spines in a row is c. 10 μm, and they vary between 2 to 35 μm in length and 1 to 6 μm in width. The base ornamentation appears to be equivalent to the rest of the surface.

**Remarks**: *Ramochitina boliviensis* Grahn, 2002 [7] differs from *Ramochitina ramosi* (Sommer & van Boekel, 1964) [74] by its longer, more sparsely distributed and variable spines, and in addition in *R*. *ramosi* the spines are always bifurcated. *R*. *boliviensis* may display different types of ornamentation in one specimen; the spines can be simple, bifurcated or branching several times near the base or at their tips. Additionally, on some occasions simple, stout, tapering spines can also occur over the vesicle surface.

The specimens recovered from the Tacobo borehole display the same vesicle shape, measurements and variability of spines described for the species. The badly preserved specimens were assigned to the species with some doubt. In these cases, the spines are completely broken, but the sparse alignment of the ornamentation can be observed through the attachment scars, and the vesicle shape and measurements coincide with those for *R boliviensis*.

**Occurrence:** Global distribution: *Ramochitina boliviensis* Grahn, 2002 [7] is restricted to Western Gondwana. Argentina and Bolivia: early to middle Givetian of the Huamampampa, Los Monos, and Iquiri formations [7,67], and in Brazil and Paraguay: Givetian of the São Domingos and Lima formations, Paraná basin [64].

*Ramochitina boliviensis* Grahn, 2002 [7], is recorded herein from the early Givetian in the TCB X-1001-Tacobo borehole.

*Ramochitina candelariaensis* sp. nov.

.2003 *Ramochitina perezi* nomen nudum, Paris *in* Paris et al., pl. 2, figs 8, 11 a–b, 12; pl. 4, figs 15, 16, 19. [76]

.2007 *Ramochitina candelariaensis* nomen nudum, Pérez-Leytón, pl. 29, figs 4–6.

Fig 7F–7I [9].

**Derivation of the name**: Following the original *nomen nudum* etymology described by Pérez-Leytón [9 p.172], being named after the La Candelaria section (Department of Chuquisaca, Bolivia). Note that this was following a more detailed study of the material as analyzed by Paris et al. [76].

**Type specimen**: Holotype: Fig 7F

**Material**: Eighteen specimens; eleven well preserved, and seven specimens assigned with some doubt, were observed and measured with an SEM from samples 9160 (4830 m), 9161 (4870 m), 9162 (4975 m), 9163 (5035 m), 9164 (5065 m) and 9116 (5198–5200) (Table 21).

**Table 21:** *Ramochitina candelariaensis* measurements.

Dimensions (μm)	L	ln	lc	D	dn	da	%neck	fracc	L:D	lc:ln	D:dn	L:lc
Mean	192	58	134	78	41	42	30%	1/3	2:1	2:1	2:1	1:1
Min	150	28	97	61	31	31						
Max	250	83	189	98	53	54						

**Diagnosis:** *Ramochitina* species with a subcylindrical to ovoid vesicle chamber with aligned spines which may be simple, lambda or multirooted.

**Description**: The vesicle chamber is subcylindrical to ovoid, with a broadly rounded basal margin and a convex to flat base. The flanks are slightly convex to straight, with a gentle flexure and very weak to inconspicuous shoulder. The short neck is cylindrical with a non-flaring collarette and occupies one-third (30%) of the total length. The ornamentation which covers the surface consists of densely aligned spines spaced between 2 and 5 μm. The spines may be simple, lambda or multirooted and they measure between 2 to 20 μm long and 0.5 to 4 μm in width (Fig 7F’). The base bears similar but smaller ornamentation compared with the rest of the vesicle wall.

**Remarks**: Pérez-Leytón [9 p. 296] described as diagnostic features “*Ramochitina* with cylindrical-ovoid chamber, short neck, flat bottom, ornamented by simple, thin, very densely arranged spines”. However, in the detailed description, they mentioned that the base could be either flat or slightly convex due to flattening. The dimensions of the Tacobo specimens are similar to those observed by Pérez-Leytón (vesicle size, 168 (211.69) 255 μm; neck occupying ~1/3 to 1/4 of the total of the vesicle). The slender outline and ornamentation are the main diagnostic features of this species. The variety of the spines is not mentioned as the main characteristic, but they are detailed in the description. In the material recovered herein, the variety of the spines seems to be an important characteristic and is thus considered a diagnostic feature for the taxon as formally described herein.

*Ramochitina callawayensis* (Urban & Kline, 1970) [77], resembles *Ramochitina candelariaensis* sp. nov., but they differ in the latter having a larger overall size and smaller neck (in *R*. *candelariaensis* the neck occupies 1/3–1/4 of the total length, meanwhile in *R*. *callawayensis* the neck occupies 2/5 of the total length). The size of the spines is similar; however, the ornamentation is much denser in *R*. *candelariaensis* sp. nov. *Ramochitina implicationis* (Urban, 1972) [59] differs from our species in having a cylindro-spheroid chamber shape, smaller overall size and shorter spines, which are usually bifurcated. *Ramochitina jutaiense* Grahn et al., 2003 [78], has an ovoid elongated body and the ornamentation consists only of multirooted and simple spines, meanwhile, *R*. *candelariaensis* bears more diverse ornamentation.

**Occurrence:** Global distribution: *Ramochitina candelariaensis* sp. nov. is restricted to Western Gondwana. It was described by Paris et al. [76] and Pérez-Leytón [9] from the early Givetian of the Huamampampa Formation in South Bolivia. In the Tacobo borehole, it is recorded from the early Givetian of the Los Monos Formation. The holotype is recorded in sample 9160 (4830 m).

*Ramochitina stiphrospinata* Grahn & Melo, 2005 [39]

.1985 *Gotlandochitina* sp. B; Paris et al., p. 72, pl. 28, figs 7–9. [61]

.2002 *Ramochitina* sp. B; Grahn, p. 320, fig. 5: B. [7]

.2002 *Ramochitina* sp. B; Grahn et al., p. 140, pl. 1, fig. H, L. 7, figs G–H. [64]

.2005 *Ramochitina stiphrospinata*; Grahn & Melo, p.27, L. 5, fig. 7, L. 8, fig. 1. [39]

.2015 *Ramochitina stiphrospinata*; di Pasquo et al., p.76, pl. 8, fig. 11. [67]

Fig 7J and 7K.

**Type specimen**: Holotype: Lange [79 pl. 2, fig. 15].

**Material**: Four specimens, three well-preserved and one fractured, were observed and measured from samples 9116 (5198–5200 m), 9163 (5035 m) and 9164 (5065 m) (Table 22).

**Table 22:** *Ramochitina stiphrospinata* measurements.

Dimensions (μm)	L	ln	lc	D	dn	da	%neck	fracc	L:D	lc:ln	D:dn	L:lc
Mean	143	42	100	77	41	39	30%	1/3	2:1	2:1	2:1	1:1
Min	131	31	84	74	37	34						
Max	153	52	112	82	48	48						

**Description**: The vesicle chamber is ovoid, with a broadly rounded basal margin and a convex base. The flanks are straight with a weak convexity in some specimens. The flexure is inconspicuous, and the shoulder is absent. The short neck is cylindrical with a non-flaring collarette and occupies one-third (30%) of the total length. Densely aligned spines ornament the vesicle surface (space between spines c. 5 μm). The spines may be simple or multi-branching at the base and the measurements vary between 5 to 27 μm long and 0.5 to 3 μm in width. Basal features could not be discerned due to the inward flattening of the specimens.

**Remarks:** The specimens assigned herein to *Ramochitina stiphrospinata* Grahn & Melo, 2005 [39], have the same vesicle outline and ornamentation characteristic of the species. Grahn and Melo [39] mentioned that the specimens of *R*. *stiphrospinata* from the Parnaíba Basin are smaller than those described by Lange [79] and Grahn et al. [64] in the Paraná Basin—the material recovered herein, however, bears the same dimensions as those previously recorded for the Paraná Basin by Grahn and Melo [39].

**Occurrence:** Global distribution: *Ramochitina stiphrospinata* Grahn & Melo, 2005 [39] was recorded in Northern Gondwana from the early Givetian of Well A1-37 in Libya [61]. Western Gondwana: from the early Givetian of the São Domingos Formation, Paraná Basin, Brazil [64,79] and the early Givetian of the Pimenteira Formation, Parnaíba Basin, Brazil [39]; from the Givetian of the Huamampampa Formation, Bolivia [7]; from the Eifelian to Givetian of the Los Monos and Iquiri formations, Northern Argentina and Bolivia [67].

*Ramochitina stiphrospinata* Grahn & Melo, 2005 [39], is recorded herein from the early Givetian in the TCB X-1001-Tacobo borehole.

*Ramochitina* sp. cf. *durandii* nomen nudum Pérez-Leytón, 2007 [9]

Fig 7L.

**Material**: One well-preserved specimen was observed and measured with an SEM from sample 9163 (5035 m) (Table 23).

**Table 23:** *Ramochitina* sp. cf. *durandii* measurements.

Dimensions (μm)	L	ln	lc	D	dn	da	%neck	fracc	L:D	lc:ln	D:dn	L:lc
Mean	220	89	130	69	46	52	40%	2/5	3:1	1:1	2:1	1:1

**Diagnosis**: *Ramochitina* species with a subcylindrical vesicle chamber and multirooted spines connected at the end forming irregular ridges parallel to the longitudinal axis.

**Fig 8:**
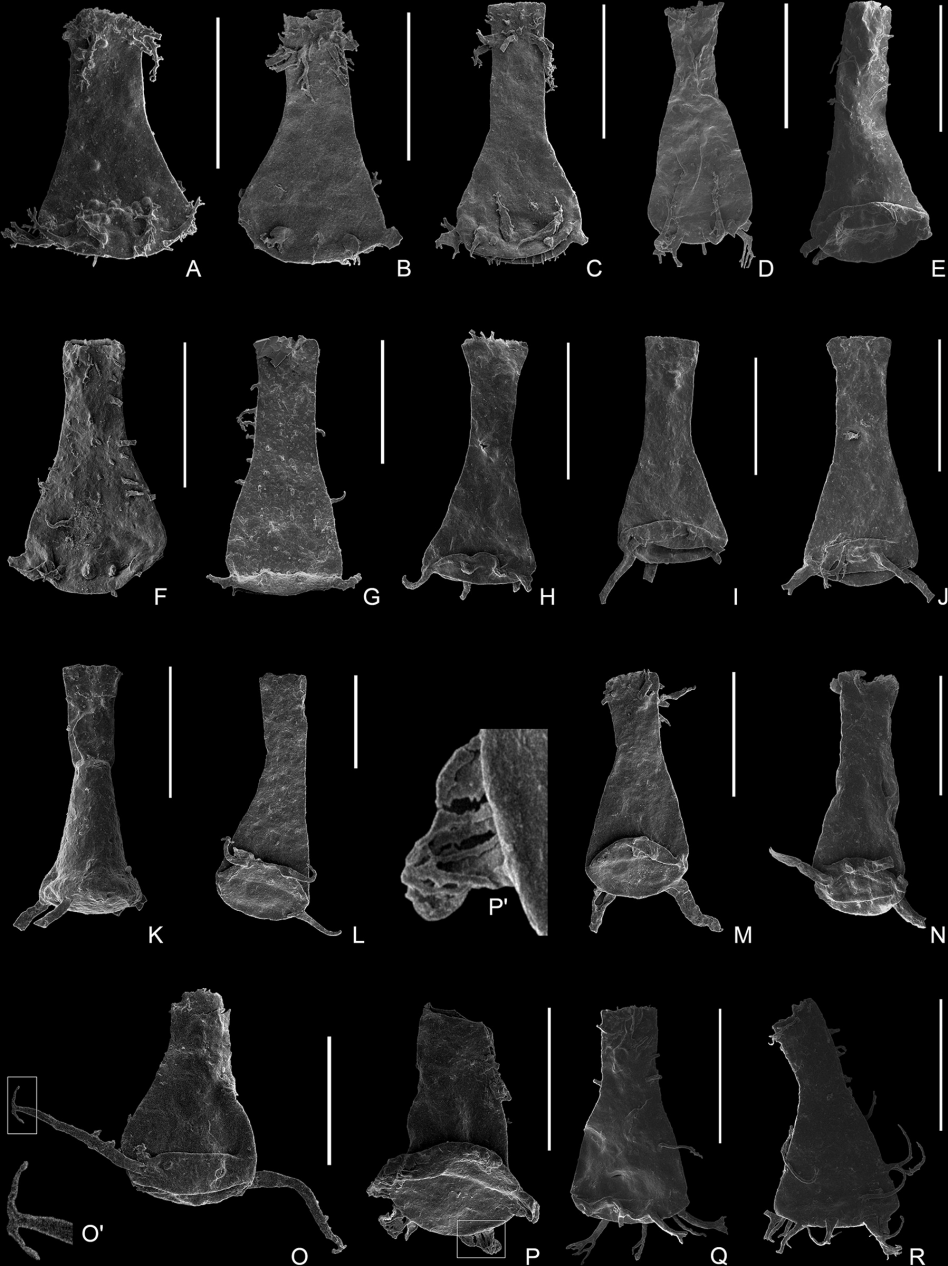
Plate 5. (A–C) *Ancyrochitina flexuosa* Almeida-Burjack, 1996, (A) 9164-S17-46, from Sample 9164; (B–C) 9162-S13-87, 9162-S10-38, from Sample 9162. (D) *Ancyrochitina* cf. *flexuosa* Almeida-Burjack, 1996, 9162-S10-18, from Sample 9162. (E) *Ancyrochitina morzadeci* Paris, 1981, 9161-S20-30, from Sample 9161. (F–G) *Ancyrochitina* aff. *morzadeci* Paris, 1981 sensu Grahn et al. (2002), (F) 9162-S10-58, from Sample 9162; (G) 9164-S3-02, from Sample 9164. (H–L) *Ancyrochitina parisi* Volkheimer et al., 1986, (H) 9163-S22-79, from Sample 9163; (I–L) 9162-S12-79, 9162-S12-37, 9162-S16-73, 9162-S13-48, from Sample 9162. (M–N) *Ancyrochitina* cf. *langei* Sommer & van Boekel, 1964, (M) 9162-S13-56, from Sample 9162; (N) 9161-S20-62, from Sample 9161. (O) *Ancyrochitina* sp. A, 9161-S19-41, from Sample 9161; (O’) detail of the processes bifurcated at their tips. (P) *Clathrochitina*? sp., 9163-S22-66, from Sample 9163; (P’) detail of the coalescent processes with a thin membrane between them. (Q–R) Alpenachitina sp. A; (Q) 9158-S21-37, from Sample 9158; (R) 9161-S20-41, from Sample 9161. All scale bars represent 100 μm.

**Description**: The vesicle chamber is sub-cylindrical, with a weakly rounded basal margin and a convex base. The flanks are slightly convex with a distinct flexure and a very weak shoulder. The neck is cylindrical and the aperture bears a simple non-flaring collarette. The ornamentation consists of birooted, multirooted and coalescent spines aligned with the axis of symmetry of the vesicle (Fig 8L’). The spines are between 7 to 18 μm long, 1 to 3 μm in width and they reach 12 μm in examples that are coalescent. The ornamentation on the base could not be discerned, as a consequence of inward flattening.

**Remarks**: *Ramochitina* cf. *durandii* n. n. bears resemblance to *R*. *durandii* n. n. Pérez-Leytón, 2007 [9] in terms of vesicle size, but they differ in the vesicle shape since the latter is elongated-conical and our specimen has a sub-cylindrical chamber. In the original description, Pérez-Leytón [9] mentioned two types of ornamentation; one over the vesicle surface, and the other one over the basal margin. Although the spines on the vesicle wall are very similar to the multirooted spines connected at the end, which form irregular ridges parallel to the longitudinal axis, described for *R*. *durandii* n. n. by Pérez-Leytón [9], the basal margin ornamentation was not observed in the Tacobo specimen. These coalescent and low-density rows of spines have not been found in other species of the genus, but due to the similarity with one of the ornamentations described for *Ramochitina durandii* n. n. it is considered here to be a species with affinity to the aforementioned taxon. Since only one specimen was recovered herein, open nomenclature is preferred for the Tacobo material.

**Occurrence:** Global distribution: *Ramochitina durandii* n. n. Pérez-Leytón, 2007 [9] is restricted to Western Gondwana. It was described from the Los Monos Formation in the Middle Givetian of south Bolivia.

*Ramochitina* sp. cf. *durandii* n. n. Pérez-Leytón, 2007 [9], is recorded herein from the early Givetian in the TCB X-1001-Tacobo borehole.

*Ramochitina* sp.

Fig 7M.

**Material**: Two moderately well-preserved specimens were observed and measured from sample 9161 (4870 m) (Table 24).

**Table 24:** *Ramochitina* sp. measurements.

Dimensions (μm)	L	ln	lc	D	dn	da	%neck	fracc	L:D	lc:ln	D:dn	L:lc
Mean	176	83	93	86	44	49	50%	1/2	2:1	1:1	2:1	2:1
Min	164	76	85	68	36	35						
Max	188	91	99	98	52	58						

**Diagnosis**: *Ramochitina* species with an ovoid vesicle chamber and small interconnected spines aligned in rows.

**Description**: The vesicle chamber is ovoid, with a broadly rounded basal margin and a convex base. The flanks are convex with a marked flexure and weak shoulder. The neck is cylindrical with a non-flaring denticulate collarette, and occupies one-half (50%) of the total length. The ornamentation consists of densely aligned small spines covering the vesicle surface (space between spines c. 4 μm). The spines may be simple or multirooted and the measurements vary between 1 to 8 μm long and 0.6 to 2 μm in width. Basal features could not be discerned due to the inward flattening of the specimens.

**Remarks**: Open nomenclature is retained for the specimens herein, due to the paucity of material, and as there are no *Ramochitina* species recorded previously bearing these small interconnected spines aligned in rows. If more examples are found through future studies, it could potentially be erected as a new species.

**Occurrence**: *Ramochitina* sp. is recorded herein from the early Givetian in the TCB X-1001-Tacobo borehole.

Subfamily **Ancyrochitininae** Paris, 1981 [27]

Genus ***Ancyrochitina*** Eisenack, 1955 [31]

Type species: *Conochitina ancyrea* Eisenack, 1931 [25]

*Ancyrochitina biconstricta* (Lange, 1949) [80]

.1949 *Conochitina biconstricta*; Lange, pp. 289–296, pl. 6, figs 1–16, pl. 7, figs 1–16, pl. 8, figs 1–9. [80]

.1967 *Cladochitina biconstricta*; Lange, pp. 77–78, pl. 2, figs 21, 23. [79]

.2002 *Spinachitina biconstricta*; Grahn, p. 322, fig. 7: F. [7]

.2002 *Spinachitina biconstricta*; Grahn et al., pp. 139–140, pl. 7, figs B–C. [64]

.2003 *Ancyrochitina biconstricta*; Paris et al., pl. 2, fig. 2; pl. 3, figs 15–17. [76]

Fig 7N–7O.

**Type specimen**: Holotype: Lange [79 pl. 9, fig. 23].

**Material**: Five well-preserved specimens, and nine assigned with a measure of uncertainty, were observed and measured from samples 9162 (4975 m), 9163 (5035 m), and 9164 (5065 m) (Table 25).

**Table 25:** *Ancyrochitina biconstricta* measurements.

Dimensions (μm)	L	ln	lc	D	dn	da	%neck	fracc	L:D	lc:ln	D:dn	L:lc
Mean	227	78	150	72	33	36	35%	1/3	3:1	2:1	2:1	2:1
Min	175	55	119	56	28	23						
Max	293	101	192	89	48	48						

**Description**: The vesicle chamber is cylindro-conical, with a well-rounded basal margin and a flat to slightly concave base. The flanks are mostly straight, though they may display a weak concavity or convexity. The neck is cylindrical with a non-flaring collarette and occupies one-third (35%) of the total length. The vesicle surface is generally smooth, but some tubercules (less than 2 μm in height) may be present sparsely. Above the basal margin, a constriction is present, which can be more evident in some specimens due to differences in flattening. The base bears a crown of simple and short processes (up to 35 μm long). Similar smaller spines are present around the neck in some specimens.

**Remarks**: The specimens recovered herein all display the characteristic vesicle shape and the constriction above the basal edge. The basal processes display some degree of damage but when they are complete, they are consistent with those of *A*. *biconstricta*. In the incomplete specimens with basal processes broken or badly preserved, the distinctive vesicle chamber outline and the characteristic constriction near the base allow for tentative assignation to this species.

There are many discussions about this species. It was originally assigned to the genus *Conochitina*, and has been placed subsequently within *Cladochitina*, *Spinachitina* and finally to *Ancyrochitina*. Lange [80] described the species as *Conochitina biconstricta* whose main distinctive characteristic is a constriction on the vesicle chamber above the base; however, he mentioned that some specimens may not display this feature due to excessive flattening. Lange [79] later created the genus *Cladochitina* in 1967 and renamed the species in question as *Cladochitina biconstricta*. The species *Ancyrochitina parisi* erected by Volkheimer et al. [81] could be referring to those specimens without the constriction over the base mentioned by Lange. For some authors, *A*. *parisi* would be a junior synonym for *A*. *biconstricta*. Grahn in Grahn et al. [64,82] divided *Ancyrochitina biconstricta* into three groups: *Ancyrochitina* sp. A, *Ancyrochitina parisi*, and *Spinachitina biconstricta*, nonetheless in a later revision from 2011 [83] he considered *Conochitina biconstricta* Lange, 1949 [80] and *Cladochitina biconstricta* Lange, 1967 [79] as synonyms of *Ancyrochitina biconstricta*. Note that the genus *Cladochitina* Lange, 1967 [79] was considered a junior synonym of *Spinachitina* Schallreuter, 1963 [84] by Paris et al. [23 p. 596], and is thus not used in current chitinozoan nomenclature.

In the Tacobo borehole, we distinguish herein specimens with and without the constriction above the base and we consider them to be two different species. Every specimen assigned to *Ancyrochitina biconstricta* herein displays this characteristic feature.

**Occurrence**: Global distribution: *Ancyrochitina biconstricta* (Lange, 1949) [80] is restricted to Western Gondwana. Brazil: Devonian of the Ponta Grossa Formation, Paraná Basin [79,80]. Bolivia: lower Devonian of the Los Monos Formation [85]; Pragian up to Upper Emsian of the Icla Formation, and early Givetian of the Iquiri Formation [7]; Middle Devonian of La Escalera section [76]; Givetian of the Iquiri Formation [8]; middle Givetian of the Los Monos Formation [9].

*Ancyrochitina biconstricta* (Lange, 1949) [80] is recorded herein from the early Givetian in the TCB X-1001-Tacobo borehole.

*Ancyrochitina cornigera* Collinson & Scott, 1958 [57]

.1958 *Ancyrochitina cornigera*; Collinson & Scott, p. 18–19, pl. 2, figs 4–5, 15–19. Text-fig. 8. [57]

.1972 *Ancyrochitina cornigera*; Urban, p. 12–13, pl. 1, figs 7–8, 10–12. [59]

.1973 *Ancyrochitina cornigera*; Urban & Newport, p. 240, pl. 1, fig. 5. [60]

.1996 *Ancyrochitina cornigera*; Almeida-Burjack, pp. 62–63, pl. 2, figs 5–11. [86]

.2000 *Ancyrochitina cornigera*; Paris et al., p. 44, pl. 2, fig. 9.[87]

.2005 *Ancyrochitina cornigera*; Grahn & Melo, p. 30, pl. 6, fig. 3. [39]

Fig 7P–7R.

**Type specimen**: Holotype: Collinson & Scott [57 pl. 2, fig. 18].

**Material**: Eight well-preserved specimens and four assigned with a measure of uncertainty were observed and measured from samples 9158 (4725 m), 9161 (4870 m), 9162 (4975 m), 9164 (5065 m) and 9166 (5170 m) (Table 26).

**Table 26:** *Ancyrochitina cornigera* measurements.

Dimensions (μm)	L	Ln	lc	D	dn	da	%neck	fracc	L:D	lc:ln	D:dn	L:lc
Mean	158	59	101	93	39	46	35%	1/3	2:1	2:1	2:1	2:1
Min	143	26	79	78	34	39						
Max	174	81	133	103	49	62						

**Description**: The vesicle chamber is conical, with a broadly rounded basal margin and a flat to slightly convex base. The flanks are generally straight but may display a weak concavity. The flexure is inconspicuous and the shoulder is absent. The neck is cylindrical and occupies one-third (35%) of the total length. The aperture is fringed by a collarette which may flare gently and the vesicle surface is smooth. The basal margin bears a crown of 6 to 8 stout simple processes of conical shape, extending up to 50 μm long, and 16 μm in width at the base. Similar spines can be distinguished around the neck in some specimens.

**Remarks**: Although some material is damaged, the specimens recovered herein possess the typical well-developed short neck and simple short processes (that widen at the base and are sharply curved at their tips) that are characteristic of this species. The Tacobo material herein resembles closely specimens described from the Cedar Valley Formation (USA) by Collinson & Scott [57], and from Brazil by Grahn and Melo [39].

Urban [59] suggested that their *Ancyrochitina megastyla* and *Earlachitina* (now *Ancyrochitina*) *latipes* (Collinson & Scott, 1958) [57], were variations of *Ancyrochitina cornigera*. As our material herein is comparable to the type material of *A*. *cornigera* Collinson & Scott, 1958 [57], we hold to this assignation. The first appearance of this species is used to define the base of the *A*. *cornigera* Interval Range Biozone of the middle Givetian [87].

**Occurrence**: Global distribution: *Ancyrochitina cornigera* Collinson & Scott, 1958 [57] was recorded in Euramerica from the USA in middle Givetian of the Cedar Valley Formation [57,59], and the early Givetian of the Wapsipinicon Formation [60]. From Canada, in the early Givetian of the Hamilton Formation, Ontario [88]. In Western Gondwana, from Brazil: Givetian of the Ponta Grossa Formation and the lower São Domingos Formation, Paraná Basin [64,86], and late Eifelian-early Givetian of the lower Pimenteira Formation, Parnaíba Basin [39].

*Ancyrochitina cornigera* Collinson & Scott, 1958 [57] is recorded herein from the early and middle Givetian in the TCB X-1001-Tacobo borehole.

*Ancyrochitina flexuosa* Almeida-Burjack, 1996 [86]

.1996 *Ancyrochitina flexuosa*; Almeida-Burjack, p. 64, pl. 2, figs 1–4. [86]

.1973 *Ancyrochitina* cf. *A*. *desmea*; Legault, p. 18–19, pl. 2, figs 1–3. [88]

.2002 *Ancyrochitina postdesmea*; Grahn, p. 323, fig. 6: G. [7]

.2002 *Ancyrochitina postdesmea*; Grahn et al., p. 320, pl. 1, fig. B. [64]

.2005 *Ancyrochitina postdesmea*; Grahn & Melo, pp. 31–32, pl. 6, figs 7–8. [39]

.2007 *Ancyrochitina yeserae* nomen nudum; Pérez-Leytón, p. 189–190, pl. 30, figs 7–9. [9]

.2008 *Ancyrochitina* cf. *A*. *postdesmea*; Grahn et al., fig. 6: H. [89]

?2016 *Ancyrochitina flexuosa*; Grahn et al., p. 359, fig. 6: M–N. [90]

Fig 8A–8C.

**Type specimen**: Holotype: Almeida-Burjack [86 pl. 2, fig. 1].

**Material**: Nine well-preserved specimens, and two assigned with uncertainty, were observed and measured from samples 9162 (4975 m) and 9164 (5065 m) (Table 27).

**Table 27:** *Ancyrochitina flexuosa* measurements.

Dimensions (μm)	L	Ln	lc	D	dn	da	%neck	fracc	L:D	lc:ln	D:dn	L:lc
Mean	170	62	105	97	41	43	40%	2/5	2:1	2:1	2:1	2:1
Min	142	29	83	81	35	37						
Max	195	98	138	113	48	47						

**Description**: The vesicle chamber is conical to subconical, with a sharply rounded basal margin and a flat to slightly convex base. The flanks are straight to slightly convex with a gentle flexure and inconspicuous to absent shoulder. The neck is cylindrical and occupies two-fifths (40%) of the total length. The aperture is surrounded by a weakly flaring simple collarette and the vesicle surface is smooth. The basal margin displays 8 to 10 complexly branching (up to 5^th^ order) processes which are bent towards the aperture. The processes are between 20 and 60 μm long and up to 10 μm in width. The neck bears similar ornamentation, although the appendages are bent towards the base.

**Remarks**: *Ancyrochitina flexuosa* Almeida-Burjack, 1996 [86] is distinguished from other *Ancyrochitina* species by its ramified processes occurring in a crown around the base. The processes are bent towards the aperture and constitute the main branch from which secondary ramifications divide subsequently up to the fifth branching order. Similar spines surround the neck, but they are bent towards the base. The specimens recovered herein bear the distinctive processes and are of the same size and shape as *A*. *flexuosa* Almeida-Burjack, 1996 [86].

This species needs further revision considering that other species have been created with the same characteristic vesicle shape and processes as *A*. *flexuosa*, but with different dimensions. Almeida-Burjack [86] originally described and measured 29 specimens from the Paraná Basin with a total length between 136.5 and 163 μm. Grahn [7] created the species *Ancyrochitina postdesmea*, from south-central Bolivia and he mentions that the main difference with *A*. *flexuosa* was the larger size, as the total length of the specimens measured therein was between 180 and 219 μm. However, Grahn and Melo [39] found smaller specimens of *A*. *postdesmea* in the Parnaíba Basin (125–140 μm long). As a consequence of this, Grahn [83] recognized that the two species were the same and he considered *A*. *postdesmea* to be a junior synonym of *A*. *flexuosa*. Another author, Pérez-Leytón [9] identified a new species, *Ancyrochitina yeserae* (nomen nudum) from the early Givetian in southern Bolivia. The main characteristic was the small vesicle size (125 to 142 μm) and the strong multi-furcated appendages at the base and around the neck. He remarked that the main difference between *A*. *yeserae* and *A*. *postdesmea* Grahn, 2002 [7] was the overall length. We consider herein that *A*. *postdesmea* and *A*. *flexuosa* are synonymous (with the latter taking precedence), and *A*. *yeserae* to be a junior synonym of this taxon (see synonymy list), as the size range fits within that described by Grahn [7] and Grahn and Melo [39] (i.e. 136.5 to 219 μm).

**Occurrence**: Global distribution: *Ancyrochitina flexuosa* Almeida-Burjack, 1996 [86] was recorded in Euramerica from Canada in the early Givetian of the Hamilton Formation, southwestern Ontario [88]. Western Gondwana: from the Givetian of the Ponta Grossa Formation, and early Givetian of the São Domingos Formation, Paraná Basin, Brazil [64,86], and from Bolivia in the early to middle Givetian of the Huamampampa and Los Monos formations [7], the latest Eifelian-earliest Givetian of the Los Monos Formation [8], and the lower to middle Givetian of the Tatarenda X27 and Camiri 201 boreholes [9].

*Ancyrochitina flexuosa* Almeida-Burjack, 1996 [86] is recorded herein from the early Givetian in the TCB X-1001-Tacobo borehole.

*Ancyrochitina* cf. *flexuosa* Almeida-Burjack, 1996 [86]

Fig 8D.

**Material**: Three moderately well-preserved specimens were observed and measured from samples 9161 (4870 m) and 9162 (4975 m) (Table 28).

**Table 28:** *Ancyrochitina* cf. *flexuosa* measurements.

Dimensions (μm)	L	ln	lc	D	dn	da	%neck	fracc	L:D	lc:ln	D:dn	L:lc
Mean	214	74	140	88	37	46	35%	1/3	2:1	2:1	2:1	2:1
Min	189	61	126	80	32	43						
Max	233	94	165	100	43	48						

**Description**: The vesicle chamber is subconical, with a broadly rounded basal margin and a flat base. The flanks are straight to gently convex, with a weakly developed flexure and inconspicuous to absent shoulder. The neck is cylindrical and occupies one-third (35%) of the total length. The aperture possesses a slightly flaring collarette that can be softly denticulated in some specimens. The vesicle surface is smooth. The basal margin bears a crown of 6 complexly multi-branching (up to 5^th^ order) processes which can reach 60 μm long and up to 8 μm in diameter. The neck holds smaller, similar ornamentation, but as a consequence of bad preservation, they are mostly fractured.

**Remarks**: The Tacobo specimens are more slender than *Ancyrochitina flexuosa* Almeida-Burjack, 1996 [86] and with spines not clearly developed. Although the material herein is scarce and highly damaged, the basal processes bear similar complex branching to *A*. *flexuosa*. The main ramification subdivides subsequently into a higher order of branching until at least the third order. However, the poor preservation of the material does not allow for the distinction of any other characteristic features, and so open nomenclature is retained for the specimens herein.

**Occurrence**: *Ancyrochitina* cf. *flexuosa* Almeida-Burjack, 1996 [86] is recorded herein from the early Givetian in the TCB X-1001-Tacobo borehole.

*Ancyrochitina morzadeci* Paris, 1981 [27]

.1981 *Ancyrochitina morzadeci*; Paris, p. 281, fig. 123, pl. 36, fig. 7, 17. [27]

.1988 *Ancyrochitina morzadeci*; Boumendjel et al., p. 340, pl. 4, fig. 8. [62]

.2002 *Ancyrochitina morzadeci*; Grahn et al., p. 139, pl. 5, fig. E. [64]

.2003 *Ancyrochitina morzadeci*; Grahn & Melo, p. 375, 384, pl. 2, fig. 7. [91]

.2005 *Ancyrochitina morzadeci*; Grahn & Melo, p. 31, pl. 6, figs 5–6. [39]

.2010 *Ancyrochitina morzadeci*; Grahn et al., p. 364, fig. 9: E. [92]

.2018 *Ancyrochitina morzadeci*; Noetinger et al., p. 103, pl. V, fig. 1–2. [75]

Fig 8E.

**Type specimen**: Holotype: Paris [27 pl. 36, fig. 7].

**Material**: One moderately well-preserved specimen, and one assigned with some uncertainty, were observed and measured from sample 9161 (4870 m) (Table 29).

**Table 29:** *Ancyrochitina morzadeci* measurements.

Dimensions (μm)	L	ln	lc	D	dn	da	%neck	fracc	L:D	lc:ln	D:dn	L:lc
Mean	241	114	127	90	39	41	50%	1/2	3:1	1:1	2:1	2:1
Min	207	90	118	86	37	34						
Max	275	138	138	93	42	48						

**Description**: The vesicle chamber is conical, with a sharply rounded basal margin and a slightly concave base. The flanks are straight with a gentle flexure. The neck is cylindrical with a non-flaring collarette and occupies one-half (50%) of the total length. The neck surface is entirely covered with simple and bifurcated spines which can reach up to 15 μm long; meanwhile, the rest of the vesicle surface is smooth. The basal margin displays 6 processes bifurcated at their tips, which measure between 15 and 40 μm long, and up to 7 μm in width near the base.

**Remarks**: Although our material is scarce and not perfectly preserved, the main characteristic features of *Ancyrochitina morzadeci* Paris, 1981 [27] can be distinguished. The specimens herein have the same vesicle shape and slender outline, and the diagnostic narrow long neck with simple spines all over the surface. The basal processes are mostly fractured, although at least one of them is complete and displays the branching nature originally described by Paris [27].

**Occurrence**: Global distribution: *Ancyrochitina morzadeci* Paris, 1981 [27] was recorded in Eastern Gondwana from the late Emsian of the Marettes Formation, France [27], and the late Emsian–Eifelian of the Illizi Basin, Algeria [62,93]. In Western Gondwana: from the early Givetian of the São Domingos Formation, Paraná Basin and early-middle Givetian of the lower Pimenteira Formation, Parnaíba Basin, Brazil [39,64], from the early Givetian of the Los Monos Formation, south-central Bolivia [7], and the Givetian of the Los Monos Formation in Angosto del Pescado, northwestern Argentina [75].

*Ancyrochitina morzadeci* Paris, 1981 [27] is recorded herein from the early Givetian in the TCB X-1001-Tacobo borehole.

*Ancyrochitina* aff. *morzadeci* Paris, 1981 [27] sensu Grahn et al. (2002) [64]

.2002 *Ancyrochitina* aff. *A*. *morzadeci*; Grahn et al., p. 139, pl. 6, figs A–B. [64]

Fig 8F–8G.

**Material**: Eight moderately well-preserved specimens were observed and measured from samples 9161 (4870 m), 9162 (4975 m), 9163 (5035 m), 9164 (5065 m) and 9166 (5170 m) (Table 30).

**Table 30:** *Ancyrochitina* aff. *morzadeci* measurements.

Dimensions (μm)	L	ln	lc	D	dn	da	%neck	fracc	L:D	lc:ln	D:dn	L:lc
Mean	177	71	106	94	42	46	40%	2/5	2:1	1:1	2:1	2:1
Min	145	50	79	81	36	40						
Max	211	112	135	100	48	53						

**Description**: The vesicle chamber is conical, with a sharply rounded basal margin and a flat to convex base. The flanks are straight to convex with a gentle flexure and inconspicuous to absent shoulder. The neck occupies two-fifths (40%) of the total length and is cylindrical with a slightly flared collarette. The vesicle surface is entirely covered with simple long spines up to 20 μm long. The basal margin bears a crown of 10 to 12 multi-branching processes that can reach 50 μm long and 10 μm in diameter.

**Remarks**: Grahn et al. [64] reported specimens of *Ancyrochitina* aff. *A*. *morzadeci* with more convex flanks and smaller neck than *Ancyrochitina morzadeci* Paris, 1981 [27], and Grahn [83] mentioned also that those specimens could possibly be a different species but that the degree of damage and paucity of material did not allow for further clarification.

Our material strongly resembles those specimens assigned to *Ancyrochitina* aff. *A*. *morzadeci* by Grahn et al. [64]. The main difference besides the shape of the vesicle chamber is the presence of the spines all over the neck and below the flexure. In *A*. *morzadeci*, the spines only are present on the surface of the neck and the rest of the vesicle chamber is smooth, only bearing the characteristic basal process over the basal margin. Another difference mentioned by Grahn [83] is the stratigraphical range since *Ancyrochitina* aff. *A*. *morzadeci* Grahn et al., 2002 [64] was recorded from the late Eifelian–middle Givetian, while *Ancyrochitina morzadeci* was reported from the late Emsian by Paris [27]. This statement would not be accurate for our material, however, since we can distinguish both species in our samples. Nonetheless, we agree with Grahn [83] that the specimens assigned to *Ancyrochitina* aff. *morzadeci* are likely a different species, but due to the scarce material and the degree of damage, open nomenclature is preferred for this species.

**Occurrence**: *Ancyrochitina* aff. *morzadeci* Paris, 1981 [27] is recorded herein from the early Givetian in the TCB X-1001-Tacobo borehole.

*Ancyrochitina parisi* Volkheimer et al., 1986 [81]

.1986 *Ancyrochitina parisi*; Volkheimer et al, pp. 236–237, fig. 6, Nr. 1–6. [81]

.2000 *Ancyrochitina parisi*; Grahn et al, p.172, pl. 5, fig. 2. [82]

.2002 *Ancyrochitina parisi*; Grahn, p.319, fig. 4I. [7]

.2011 *Cladochitina varispinosa*; Troth et al, p.7, fig. 5G. [20]

.2013 *Ancyrochitina parisi*; Noetinger & di Pasquo, p.115, fig. 6B. [94]

Fig 8H–8L.

**Type specimen**: Holotype: Volkheimer et al. [81 fig. 6, Nr. 1].

**Material**: Ten well-preserved specimens, and seventy-three displaying varying levels of damage to the processes, were observed and measured from samples 9162 (4975 m) and 9163 (5035 m) (Table 31).

**Table 31:** *Ancyrochitina parisi* measurements.

Dimensions (μm)	L	ln	lc	D	dn	da	%neck	fracc	L:D	lc:ln	D:dn	L:lc
Mean	193	78	115	88	37	43	40%	2/5	2:1	1:1	2:1	2:1
Min	150	50	82	69	32	20						
Max	271	119	183	127	54	67						

**Description**: The vesicle chamber is conical to sub-ovoid, with a broadly rounded basal margin and a generally flat to slightly convex base. The flanks are straight, but they may display a weak convexity or concavity. The flexure is weakly developed and the shoulder is inconspicuous or absent. The neck is cylindrical and occupies two-fifths (40%) of the total length. The aperture is enclosed by a thin-walled slightly flaring collarette that in some specimens can be denticulated. The vesicle surface is smooth and the basal margin bears a crown of 6 to 8 simple processes (up to 80 μm long and 15 μm in diameter). The neck has similar ornamentation with spines that can reach 15 μm long.

**Remarks**: *Ancyrochitina parisi* Volkheimer et al., 1986 [81], is very similar to *Ancyrochitina biconstricta* Almeida-Burjack, 1996 [86], the main difference being the absence of a marked constriction above the base (see discussion in *A*. *flexuosa*). The original description of this species mentions that constriction can be present in some of the specimens, but this feature seems to be a consequence of the difference in flattening and not an inherent characteristic of the species. *A*. *parisi* also differs from *A*. *biconstricta* in the nature of the basal processes which in the former can not only be simple but also bifurcated and usually more robust. Although our material is flattened and mostly damaged, diagnostic features are always noticeable in all of the specimens, in particular the very distinctive vesicle outline.

*Ancyrochitina parisi* Volkheimer et al., 1986 [81], was originally described from the uppermost Lower Devonian in the Puesto El Tigre Formation. The age was estimated because the strata, which contained this species, were lying immediately above those with *Ramochitina magnifica* and below those with *Sphaerochitina pilosa* (= *Fungochitina pilosa*). These authors proposed a local biozonation with *Ancyrochitina parisi* as an index species from the uppermost Lower Devonian. Grahn et al. [64,82], Grahn [7,10], and Noetinger and di Pasquo [66,94], recorded this species from the late Emsian, possibly including the earliest Eifelian, from Brazil, Bolivia and northern Argentina. In the Western Gondwanan biozonation proposed by Grahn [10], the first occurrence of *A*. *parisi* defines the beginning of the *Ancyrochitina parisi* Interval Range Zone for the late Emsian. The absence of this species in younger strata from Western Gondwanan basins defined the previously restricted stratigraphical range. These new records from Tacobo would extend the range of *A*. *parisi* in southern Bolivia to the early Givetian. Nevertheless, there is one record from Troth et al. [20] in Bolivia where it is described an acme of the chitinozoan *Cladochitina varispinosa* (Lange, 1967) [79] in the middle part of Los Monos Formation (p. 7, fig. 5G). This record could be the same one that we observe from the middle part of the Tacobo borehole since the main characteristics of the *C*. *varispinosa* species are not seen and both vesicle outline and processes seem to be like those from *A*. *parisi*. In this case, there would be a previous record from the early Givetian for *A*. *parisi* in southern Bolivia.

**Occurrence**: Global distribution: *Ancyrochitina parisi* Volkheimer et al., 1986 [81] is restricted to Western Gondwana. Northwestern Argentina: late Emsian of the Puesto El Tigre Formation and San Antonio x-1 borehole [66,81,94]. Southern Brazil: late Emsian of the Ponta Grossa Formation, Paraná Basin [82]. Southern Bolivia: late Emsian-earliest Eifelian of the Icla Formation [7], and the early Givetian of the Los Monos Formation [20].

*Ancyrochitina parisi* Volkheimer et al., 1986 [81] is recorded herein from the early Givetian in the TCB X-1001-Tacobo borehole.

*Ancyrochitina* cf. *langei* Sommer & van Boekel, 1964 [74]

1964 *Ancyrochitina langei*; Sommer & van Boekel, p. 427, pl. 1, fig. 1, text-fig. 4. [74]

1967 *Ancyrochitina langei*; Lange, p. 70, pl. 1, figs 8–9. [79]

1982 *Ancyrochitina langei*; Quadros, pp. 40–41, pl. 1, fig. 4. [95]

1996 *Ancyrochitina langei*; Almeida-Burjack, p. 66, pl. 4, fig. 5. [86]

2002 *Ancyrochitina langei*; Grahn et al., pp. 140, 148, pl. 8, figs B–E. [64]

2003 *Ancyrochitina langei*; Grahn et al., p. 294, pl. 4, fig. 5, pl. 5, fig. 7. [78]

2005 *Ancyrochitina langei*; Grahn & Melo, pp. 30–31, pl. 8, figs 2–3. [39]

2008 *Ancyrochitina langei*; Grahn et al., p. 140, fig. 6: C–G. [89]

2010 *Ancyrochitina langei*; Grahn et al., p. 364, fig. 9: A–B. [92]

Fig 8M and 8N.

**Type specimen**: Holotype: Sommer & van Boekel [74 pl. 1, fig. 1].

**Material**: Sixteen damaged specimens were observed and measured from samples 9160 (4830 m), 9161 (4870 m) and 9162 (4975 m) (Table 32).

**Table 32:** *Ancyrochitina* cf. *langei* measurements.

Dimensions (μm)	L	ln	lc	D	dn	da	%neck	fracc	L:D	lc:ln	D:dn	L:lc
Mean	193	61	132	82	41	44	30%	1/3	2:1	2:1	2:1	1:1
Min	169	47	102	69	33	33						
Max	246	76	180	94	48	55						

**Description**: The vesicle chamber is subcylindrical to conical, with a well-rounded to sharply rounded basal margin and a slightly convex base. The flanks are straight to gently convex with a flexure and shoulder that are inconspicuous. The neck occupies one-third (30%) of the total length and is cylindrical with a thin-walled slightly denticulated collarette around the aperture. The vesicle surface is generally smooth nonetheless; the body or the neck may bear some small randomly distributed tubercules (less than 2 μm in height). A crown of 6 to 8 simple long processes (mostly broken) can be recognized over the basal margin with measurements between 30 to 80 μm long and a diameter between 6 and 13 μm near the base. Broken processes and scars of similar nature are recognized around the neck in all specimens.

**Remarks**: The Tacobo borehole specimens assigned to *Ancyrochitina* cf. *langei* are mostly poorly preserved. Basal processes can be discernible, but most of them are fractured or completely missing. The slender vesicle outline and short cylindrical neck bear a strong resemblance to *A*. *langei* Sommer & van Boekel, 1964 [74]. The basal processes, when they are complete, display similar measurements and shape to those described for *A*. *langei*, and as such the main differentiation is the slightly bigger vesicle size of *A*. cf. *langei* herein. Previous records of *A*. *langei* from Bolivia [7] display strong similarities to our *A*. cf. *langei* material, even having the same degree of damage since broken processes are very common. Nonetheless, an open nomenclature is preferred for the Tacobo material.

**Occurrence**: Global distribution: *Ancyrochitina langei* Sommer & van Boekel, 1964 [74] was recorded in Euramerica from Canada: early Givetian of the Hamilton Formation, southwestern Ontario [88]. Western Gondwana, from Brazil: Devonian of the ‘locality 24’ outcrops at Tocantinia, Goiás [74]; Middle Devonian of the São Domingos Formation [79]; Givetian of the Ponta Grossa Formation [86]; and late Eifelian of the Chapada Group, Paraná Basin [64,92]; early Givetian and Emsian-Eifelian of the Biá, Jandiatuba and Uêre formations, Solimões Basin [78,95]; late Eifelian-middle Givetian of the Itaim and Pimenteira formations [39]; early Givetian of the Pimenteira Formation, Parnaíba Basin [89]. From Bolivia: early-middle Givetian of the Huamampampa and Los Monos formations [7]; middle-late Givetian of the Iquiri Formation [8].

*Ancyrochitina* cf. *langei* Sommer & van Boekel, 1964 [74] is recorded herein from the early Givetian in the TCB X-1001-Tacobo borehole.

*Ancyrochitina* sp. A.

Fig 8O.

**Material**: One well-preserved specimen was observed and measured from sample 9161 (4870 m) (Table 33).

**Table 33:** *Ancyrochitina* sp. A. measurements.

Dimensions (μm)	L	ln	lc	D	dn	da	%neck	fracc	L:D	lc:ln	D:dn	L:lc
Mean	171	45	126	105	46	-	25%	1/4	3:1	2:1	2:1	1:1

**Diagnosis**: *Ancyrochitina* species with an ovoid vesicle chamber and very long processes which bifurcate delicately at their tips.

**Description**: The vesicle chamber is ovoid, with a broadly rounded basal margin and a flat to slightly convex base. The flanks are convex with a marked flexure and a distinct shoulder. The neck is cylindrical, but the aperture is fractured and no other features could be discerned. The vesicle surface is smooth and over the basal margin, a crown of 6 long processes can be distinguished, which are up to 115 μm in length and delicately bifurcated at their tips (Fig 8O’).

**Remarks**: The specimen recovered herein is broken over the aperture, but the vesicle chamber features can be distinguished clearly. The ovoid vesicle shape is uncommon in the genus *Ancyrochitina*, but the presence of processes on the basal margin suggests this generic assignment. The long, thin, distally-bifurcated processes are not seen in any other *Ancyrochitina* species. Taxa belonging to the genus *Plectochitina* bear similar types of appendices, but they also should display a spongy texture that is not apparent in our specimen. Based only upon on a single broken specimen bearing these characteristics, it is retained in open nomenclature.

**Occurrence**: *Ancyrochitina* sp. A Is recorded herein from the early Givetian in the TCB X-1001-Tacobo borehole.

*Ancyrochitina* sp. indet.

**Material**: Seventy-six badly preserved specimens were observed from samples 9158 (4725 m), 9161 (4870 m) and 9162 (4975 m).

**Description**: All the material bears broken remnants or scars where the processes were attached. The vesicles are generally broken and without a distinguishing outline that would allow for assignation to species level. The vesicle surface of all specimens is smooth without any ornamentation. No other features could be discerned.

**Remarks**: The specimens can be assigned with confidence to *Ancyrochitina*, based upon the presence of basal processes or attachment scars on the basal margin. All specimens bear a smooth vesicle surface and no diagnostic vesicle shape. Due to a lack of any further diagnostic features other than the basal processes, identification to species level could not be attempted.

Genus ***Clathrochitina*** Eisenack, 1959 [29] emend. Laufeld, 1974 [69]

Type species: *Clathrochitina clathrata* Eisenack, 1959 [29]

*Clathrochitina*? sp.

Fig 8P.

**Material**: One flattened and broken specimen was observed and measured from sample 9163 (5035 m) (Table 34).

**Table 34:** *Clathrochitina*? sp. measurements.

Dimensions (μm)	L	ln	lc	D	dn	da	%neck	fracc	L:D	lc:ln	D:dn	L:lc
Mean	163	-	-	96	49	-	-	-	2:1	-	2:1	-

**Diagnosis**: Ancyrochitininae with a subcylindrical vesicle chamber with anastomosed basal process with a membrane in between.

**Description**: The vesicle chamber is subcylindrical, with a sharp basal margin and concave base. The flanks are straight and the flexure and shoulders are absent. The length of the neck cannot be discerned due to the broken aperture and the vesicle surface is smooth. The basal margin displays a crown of 6 coalescent processes with a thin membrane between them (Fig 8P’).

**Remarks**: Only one broken specimen could be assigned with uncertainty to the genus *Clathrochitina* based upon the nature of the basal processes. This specimen bears a thin membrane between the processes which is not present in *Clathrochitina*. Another difference are the processes that should be anastomosed, as on the specimen herein they are only coalescent near the basal margin. These two unusual features make assignation to *Clathrochitina* questionable. However, this is the only genus to date which is described with complex processes, and thus the only one we reasonably make an assignation to. If further similar specimens are found, it may be possible that a new genus is erected to contain specimens with this unusual combination of features.

**Occurrence**: *Clathrochitina*? sp. is recorded herein from the early Givetian in the TCB X-1001-Tacobo borehole.

Genus ***Alpenachitina*** Dunn & Miller, 1964 [96]

Type species: *Alpenachitina eisenacki* Dunn & Miller, 1964 [96]

*Alpenachitina* sp. A.

Fig 8Q and 8R.

**Material**: Two well-preserved specimens were observed and measured from samples 9158 (4725 m) and 9161 (4870 m) (Table 35).

**Table 35:** *Alpenachitina* sp. A. measurements.

Dimensions (μm)	L	ln	lc	D	dn	da	%neck	fracc	L:D	lc:ln	D:dn	L:lc
Mean	167	69	98	93	40	40	40%	2/5	2:1	1:1	2:1	2:1
Min	162	64	97	89	37	37						
Max	173	74	99	96	42	42						

**Diagnosis**: *Alpenachitina* species with a conical vesicle chamber, with multi-branching processes aligned in two rows and the neck covered with simple spines.

**Description**: The vesicle chamber is conical, with a sharply rounded basal margin and a flat to slightly convex base. The flanks are straight with a gentle convexity and the flexure and shoulder are weakly developed to inconspicuous. The neck occupies two-fifths (40%) of the total length and is cylindrical with a non-flaring and slightly denticulated collarette. The vesicle surface is smooth. The ornamentation consists of 2 rows of processes, one over the basal margin and the other one below the shoulder. The processes are long (up to 50 μm) and mostly multi-branching at their tips, while some of them may bifurcate near the base. The neck is entirely covered with simple and branching spines.

**Remarks**: The specimens herein bear the main characteristic of the genus *Alpenachitina*, in that they possess processes and spines arranged in three well-differentiated rows. However, the nature of these processes and the vesicle shape is different to any other *Alpenachitina* species.

*Alpenachitina* sp. A has a conical vesicle chamber with a non-flaring neck, meanwhile *A*. *eisenacki* Dunn & Miller, 1964 [96], *A*. *matogrossensis* and *A*. *petroviensis* Almeida-Burjack & Paris, 1989 [97], have cylindrical to ovoid vesicle chambers; *A*. *crameri* Hutter, 1979 [98], has an ovoid chamber with a short neck; and *A*. *ontariensis* Legault, 1973 [88], has a conical chamber but shorter flaring neck.

*A*. *matogrossensis* and *A*. *petroviensis* have elongated and tubular processes with different branching at their tips and coalescent processes, and *A*. *eisenacki* bears stout multi-branching spines. The processes upon our specimens differ in that all of them are either simple or show less complex multi-branching at their tips. Due to the paucity of specimens they are retained in open nomenclature.

**Occurrence**: *Alpenachitina* sp. is recorded herein from the early and middle Givetian in the TCB X-1001-Tacobo borehole.

## 5. Discussion

### 5.1. Definition of biozones

**Fig 9:**
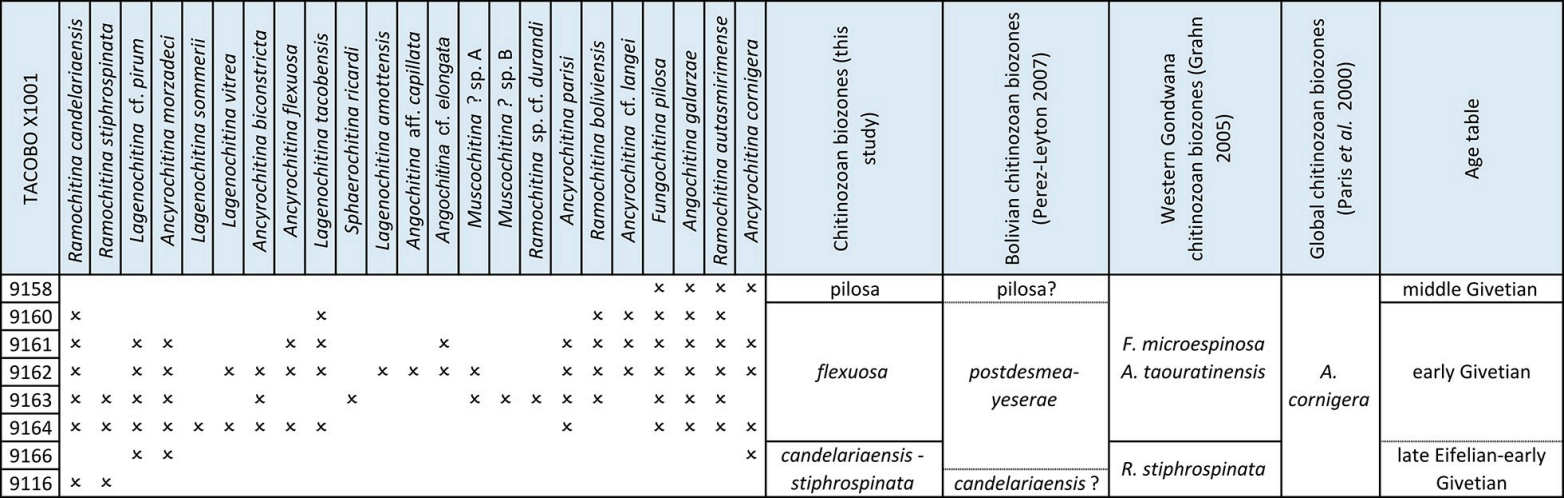
Chitinozoan biozonation. Proposed chitinozoan biozonation for the TCB X-1001-Tacobo borehole and the correlation with Local, Regional and Global chitinozoan biozonations.

Chitinozoan biozonations are usually proposed over well studied sections as interval zones. When the material studied comes from outcrops and drilling cores, the base of the biozones are stablished with the first occurrence (FAD) of and index species until the FAD of another index species [4,87]. This is because the occurrence of the specimens are certain to be precise and not to be material coming from other stratigraphical levels. When we work with cutting material the occurrence of the specimens may be from the bearing stratigraphical level or may be from some younger beds due to the caving process from the drilling. In that case, for biostratigraphical studies the last appearance (LAD) of an index species are usually used instead of the FAD. However, if we only use the LAD, the difficulty relies on the comparison and correlation with the currently existing biozones. Therefore, we decided to treat our material with the same methodology that is used for other biozonations, knowing that the limits proposed for our biozones may be not precise and may need further adjustment in the future (Fig 9).

#### 5.1.1 The *Ramochitina candelariaensis*-*stiphrospinata* Local Assemblage Biozone

The *Ramochitina candelariaensis* Local Assemblage Biozone was originally described by Pérez-Leytón [9] as an almost monospecific biozone together with *Ancyrochitina* sp. aff. *A*. *biconstricta*. In the Tacobo borehole this biozone can be recognised in the lowermost samples (9116–9166) and is characterized by the FAD of *Ramochitina candelariaensis* sp. nov. and *Ramochitina stiphrospinata* until the FAD of *Ancyrochtina flexuosa*.

Age: late Eifelian–early Givetian.

#### 5.1.2 The *Ancyrochitina flexuosa* Local Assemblage Biozone

This biozone was originally described as the *Ancyrochitina postdesmea*-*Ancyrochitina yeserae* n. n. Local Assemblage Biozone [9]. It is characterised by the simultaneous occurrence of *Ancyrochitina postdesmea* and *Ancyrochitina yeserae* n. n. Other taxa present in the lower part of this biozone are *Ancyrochitina arirambaensis*, *Ancyrochitina biconstricta*, *Ramochitina boliviensis* and *Ramochitina devonica*, and *Ancyrochitina langei* and *Ramochitina autasmirimense* for the upper part of this biozone.

In the Tacobo borehole this biozone is erected as the *Ancyrochitina flexuosa* Local Assemblage Biozone, and can be recognised from sample 9164 until sample 9160. It is characterised by the FAD of *Ancyrochtina flexuosa* (synonymised with *Ancyrochitina postdesmea* and *Ancyrochitina yeserae* n. n.) until the LAD of *Ramochitina boliviensis*. Species such as *Ancyrochitina biconstricta*, *Ancyrochitina cornigera* and *Lagenochitina tacobensis* have also their FAD in this biozone.

Age: early Givetian.

#### 5.1.3 The *Fungochitina pilosa* Local Assemblage Biozone

Erected by Pérez-Leytón [9], this biozone for the middle-late Givetian is characterised by the presence of *Fungochitina pilosa* and a variety of *Ancyrochitina* species such as *Ancyrochitina biconstricta*, *Ancyrochtina monosi* n. n., *Ancyrochitina postdesmea*, *Ancyrochtina yeserae* n. n., and *Ancyrochitina* cf. *A*. *taouratinensis*.

In the Tacobo borehole this biozone is recognised in the uppermost sample (9151) and is characterised by the presence of *Fungochitina pilosa* and the LAD of *Ramochitina boliviensis*. Species such as *Angochitina galarzae* and *Ramochitina autasmirimense* can also be recognised in this biozone.

Age: middle Givetian.

### 5.2. Age and correlation (Fig 10)

**Fig 10:**
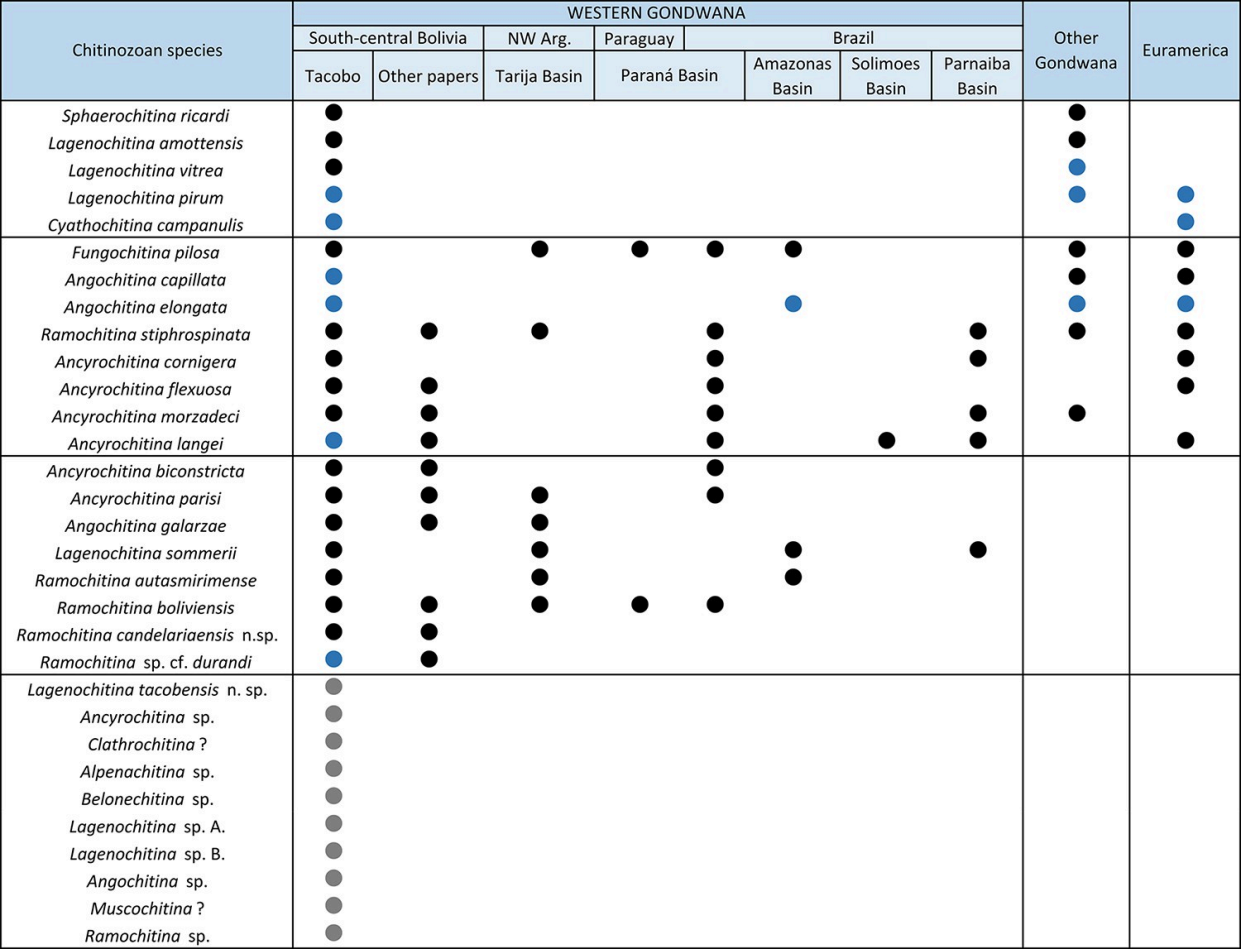
Chitinozoan ocurrences in the TCB X-1001-Tacobo borehole. The black circles represent positively identified species and the blue circle open nomenclature species. The gray circles are species restricted to the TCB X-1001-Tacobo borehole. The first group of species have their first record in Western Gondwana. The second group are species recorded in all the palaeocontinents, and the last group represents species restricted to Western Gonwana.

The Los Monos Formation is considered to be a diachronous unit as a consequence of the physiography of the basin [2]. This is inferred by the discrepancy in age proposed by different authors. In South Central Bolivia an early to middle Givetian age for the lower part of the Los Monos Formation extending to the early Frasnian at the top was proposed by Grahn [7]. In the Central South Subandean of Bolivia, an early to late Eifelian age was suggested by Troth et al. [20]. In the Sub-Andean of Bolivia and Argentina, the age inferred was late Eifelian-early Frasnian [67] and later restricted to late Eifelian-early middle Givetian [19]. For the TCB X-1001-Tacobo borehole, García Muro et al. [3] proposed an age of Eifelian?–Early Givetian to the Middle Givetian for the Los Monos Formation based on miospores and organic-walled phytoplankton, coinciding with the age proposed herein.

Although, it does not provide a tighter constraint for the dating of this unit. Coincidently with the study therein, the stratigraphical distribution of the chitinozoans was analysed based on the last appearance data (LAD) from the bottom to the top of the borehole since the palynological assemblage comes from cutting samples.

*Lagenochitina* cf. *pirum*, *Lagenochitina vitrea*, *Angochitina* cf. *elongata* and *Cyathochitina* cf. *campanulis* are species that have not previously been recorded from Western Gondwana. *L*. *pirum sensu stricto* has only been recorded previously from the Ordovician [42,48,50–53] and *L*. *vitrea* [43–45], *A*. *elongata* [25,69,71] and *C*. cf. *campanulis* [54] from the Silurian. Due to the scarcity of specimens recovered from the TCB X-1001-Tacobo borehole and differences with the type material there is no certainty that the occurrences herein would extend the range of these particular species into the middle Devonian.

*Lagenochitina sommeri* is restricted to Western Gondwana, ranging throughout the Devonian [30,37–39], and the specimens assigned to *Angochitina capillata* from the middle Devonian are from Euramerica and Iberia [33,60].

The stratigraphical range of the *Sphaerochitina ricardi* and *Ancyrochitina morzadeci* coincides with the early Givetian age of the bearing samples (9166 to 9161). The *A*. *morzadeci* early Givetian records are from Western Gondwana [7,39,64], though herein is the first record of *S*. *ricardi* for Western Gondwana.

Even though *Ramochitina durandii* is middle Givetian in age [9], it is assigned with some reservation to the early Givetian in the Tacobo borehole. *Angochitina galarzae* has a wide stratigraphical range from the Givetian to the early Frasnian [30,66,67] which coincides with the early and middle Givetian assigned to the TCB X-1001-Tacobo borehole samples (9164 to 9158).

*Ancyrochitina parisi* is restricted to Western Gondwana and was originally described in the uppermost Lower Devonian of northern Argentina [81]. Its stratigraphical range is currently restricted to the late Emsian-earliest Eifelian [7,66,82,94] and the first occurrence of this species defines the beginning of the *Ancyrochitina parisi* Interval Range Zone for the late Emsian in the Western Gondwanan chitinozoan biozonation [10]. Considering Troth et al.’s [20] record of *C*. *varispinosa* as *A*. *parisi* (see systematic discussion of the species), the last occurrence of this taxon would be in the early Givetian.

The species mentioned above are not useful to further constrain the age of the samples from the Tacobo borehole. Nevertheless, if taxa such as *Ramochitina candelariaensis* and *Ramochitina stiphrospinata* in the lowermost part of the borehole (9116–9166) and specimens of *Ancyrochitina flexuosa* in sample 9164 were not caved, this part could be assigned to the *candelariaensis*-*stiphrospinata* Biozone. This biozone correlates partially with the monospecific *candelariaensis* Bolivian local biozone proposed by Pérez-Leytón [9]. The absence of *Ramochitina candelariaensis* in sample 9166 would suggest a possible *postdesmea*-*yeserae* Biozone age for this sample. However, the Tacobo *candelariaensis*-*stiphrospinata* Biozone is characterised by the presence of both species until the FAD of *Ancyrochitina flexuosa* (*A*. *postdesmea*-*A*. *yeserae*) which coincides with the *Ramochitina stiphrospinata* Total Range Zone for Western Gondwana [10]. In this case, samples 9116–9166 could be assigned to the late Eifelian-early Givetian and would agree with the phytoplankton and miospore age proposed for this part of the borehole by García Muro et al. [3].

The presence of *A*. *biconstricta*, *A*. *flexuosa* and *R*. *boliviensis* would correspond to the early Givetian *flexuosa* local biozone which correlates partially with the *postdesmea*-*yeserae* Bolivian local biozonation [9]. *A*. *postdesmea* and *A*. *yeserae* are considered synonyms of *A*. *flexuosa* and this could explain why they are usually found associated.

The *flexuosa* biozone can be correlated with the *Fungochitina microespinosa*—*Ancyrochitina taouratinensis* from the Western Gondwana biozonation [10], however, it also could have some species in common with the *R*. *stiphrospinata* biozone according to Pérez-Leytón [9].

*A*. *biconstricta* was proposed as the index species of the middle Givetian *A*. *biconstricta* Local Assemblage Biozone of Bolivia [9]. Nevertheless, it is mentioned that this biozone is difficult to correlate with others since the species need revision (see discussion for *A*. *biconstricta*). Therefore, this taxon should not be used as an index species, and consequently it is not used herein.

*Fungochitina pilosa* is a common Middle-Late Devonian species with worldwide distribution [57–60,62,64–66]. In Western Gondwana, this species has been used together with *Ancyrochitina langei* in the Paraná Basin as the main species of a concurrent range zone for the late Givetian [64]. In the Bolivian biozonation it is also considered the index species of the *Fungochitina pilosa* Local Assemblage Biozone for the middle to late Givetian [9].

In the Tacobo borehole the occurrence of *F*. *pilosa* and the LAD of *Ramochitina boliviensis* in sample 9160 would suggest a *pilosa* biozone and possible middle Givetian age for the uppermost 9158 sample.

*Ancyrochitina cornigera* is a well-known species with worldwide distribution used by Paris et al. [87] to define the base of the middle Givetian in a global biozonation. However, it has been recorded not only in the middle Givetian [57,59] but also from the early Givetian [60,88] and late Eifelian-early Givetian [39]. Therefore, the presence of *A*. *cornigera* in the Tacobo borehole is not useful to constrain the age of the studied samples.

The presence of *Ancyrochitina biconstricta*, *Ancyrochitina parisi*, *Angochitina galarzae*, *Lagenochitina sommerii*, *Ramochitina autasmirimense* and *Ramochitina boliviensis* indicate Western Gondwana affinities (Fig 10). Only five species are recorded herein for the first time in Western Gondwana, and three of them are kept in open nomenclature.

The chitinozoan assemblage in the Tacobo borehole shows strong similarity with other assemblages from Bolivia, Paraná Basin in Brazil, and Northwestern Argentina, and can be assigned with certainty to the early Givetian (*postdesmea*-*yeserae* Biozone). If the presence of the index species of the Eifelian-early Givetian *candelariaensis* Biozone and the middle Givetian *pilosa* zone were not caved, they could extend the age of the studied interval from the late Eifelian-early Givetian to the middle Givetian. Even though the local Bolivian biozonation proposed by Pérez-Leytón [9] is not formally established, it is evident that it is more useful for this part of the basin than the Western Gondwana biozones [10]. Therefore, a review of the Western Gondwana zonation proposed by Grahn [10] is necessary, in order to be more suitable for the Bolivian basins. However, the difference in the assemblages and index species between both biozonations would suggest that some species are endemic to Bolivia and the possible existence of some physical barrier between Bolivia and the rest of Western Gondwana during this part of the Devonian.

## 6. Conclusions

1. The chitinozoan assemblage from the TCB X-1001-Tacobo borehole yielded eleven genera and thirty-five chitinozoan species, with nineteen retained in open nomenclature. *Sphaerochitina ricardi*, *Lagenochitina amottensis*, *Lagenochitina vitrea*, *Lagenochitina* cf. *pirum* and *Cyathochitina* cf. *campanulis* are recorded for the first time in Western Gondwana. Species such as *Ancyrochitina biconstricta*, *Ancyrochitina parisi*, *Angochitina galarzae*, *Lagenochitina sommerii*, *Ramochitina autasmirimense* and *Ramochitina boliviensis*, which are restricted to Western Gondwana, support the established affinity with this palaeocontinent.
2. *Lagenochitina tacobensis* sp. nov. and *Ramochitina candelariaensis* sp. nov. (previously a *nomen nudum*) are formally erected herein.
3. The stratigraphical range of *Ancyrochitina parisi* is extended from the late Emsian-earliest Eifelian to the early Givetian, and the LAD of *Ramochitina autasmirimense* from the early Givetian to the middle Givetian.
4. A new local chitinozoan biozonation based on the TCB X-1001-Tacobo borehole assemblages is proposed. For the late Eifelian–early Givetian: The *Ramochitina candelariaensis*-*stiphrospinata* Local Assemblage Biozone. For the early Givetian: The *Ancyrochitina flexuosa* Local Assemblage Biozone. For the middle Givetian: The *Fungochitina pilosa* Local Assemblage Biozone.
5. The Los Monos chitinozoan assemblage in the TCB X-1001-Tacobo borehole can be assigned to the late Eifelian-early Givetian *candelariaensis*-*stiphrospinata* Local Assemblage Biozone, the early Givetian *flexuosa* Local Assemblage Biozone, and the middle Givetian *pilosa* Local Assemblage Biozone from the youngest to the oldest records. This age coincides with the one proposed for the Los Monos Formation in the TCB X-1001-Tacobo borehole by García Muro et al. [3].
6. The current chitinozoan biozonation for Western Gondwana [10] should be revised, taking into account the Bolivian chitinozoan biozonation [9] and the new biozonation proposal based on the present study.

## Supporting information

S1 Table Tacobo X1101 Morphometrics. Morphometric dataset of Tacobo X1001 borehole chitinozoans.
(XLSX)
